# A New Paradigm for Sustainable Food Packaging: Construction, Properties, and Applications of Multifunctional Hydrogels

**DOI:** 10.3390/gels12090847

**Published:** 2026-09-16

**Authors:** Jin Yao, Zhen Cao, Tian Zhao

**Affiliations:** School of Packaging Design and Art & School of Materials Science and Engineering, Hunan University of Technology, Zhuzhou 412007, China

**Keywords:** hydrogel, food packaging, sustainable materials, biodegradation

## Abstract

Conventional petroleum-based plastic packaging is facing formidable challenges in terms of both environmental sustainability and precise quality regulation of food products, compelling the food packaging industry to pivot toward biodegradable, recyclable, and intelligent solutions. Hydrogels, characterized by their unique three-dimensional network architecture, high water content, and excellent biocompatibility, have emerged as promising candidates for constructing next-generation sustainable food-packaging systems. This review presents a structured critical overview of the recent progress in multifunctional hydrogels derived from natural and synthetic polymers for food packaging applications. We begin by summarizing the structural characteristics and functional attributes of various polymeric substrates from different sources. Subsequently, we place emphasis on elucidating the molecular design and microstructural engineering strategies that enable enhanced mechanical properties, confer antimicrobial and antioxidant activities, and impart pH/gas-responsive intelligent functionalities. Furthermore, the regulatory mechanisms of forming and processing technologies—including solution casting, electrospinning, and three-dimensional printing—on the structure and performance of hydrogel-based packaging materials are discussed in depth. Finally, in view of the key bottlenecks currently impeding the widespread adoption of hydrogel packaging materials, particularly regarding water resistance, scalable production, and cost control, we outline future directions toward multifunctional synergism and quantitatively evaluated life-cycle performance. This review aims to provide a systematic theoretical basis and technical reference for the rational design of high-performance, intelligent, and sustainable food packaging systems for the future.

## 1. Introduction

With consumers’ increasing demands for food quality and growing environmental awareness, higher requirements have been placed on food packaging materials. Food packaging materials must not only provide basic physical protection, but should also perform multiple functions, including extending food shelf life, monitoring food quality in real time, and reducing environmental pollution. However, petroleum-based synthetic plastics that have long dominated the market, such as polyethylene terephthalate (PET), polypropylene (PP), polyethylene (PE), polycarbonate (PC), polyethylene naphthalate (PEN), polystyrene (PS), polyvinyl chloride (PVC), ethylene-vinyl alcohol (EVOH), and polyamide (PA), are low in cost and possess excellent mechanical properties [1]. However, their persistence after disposal and the complexity of mixed-material packaging streams have intensified concerns regarding waste accumulation, resource consumption, and environmental impacts [2].

Hydrogels are materials with a three-dimensional network structure formed by the physical or chemical crosslinking of hydrophilic polymer chains [3]. Their unique high water content, tissue-like characteristics, and tunable physicochemical properties give them distinctive advantages in food packaging [4]. Compared with conventional passive films, hydrogel-based materials can provide hydrated networks for the incorporation, retention, and controlled release of antimicrobial agents, antioxidants, or sensing compounds. Their environmental degradation behavior depends on polymer chemistry, crosslinking, additives, processing history, and disposal conditions [5,6,7]. Accordingly, the potential advantages of hydrogel-based packaging should be evaluated in relation to the formulation, intended food-contact conditions, and relevant end-of-life pathway. The development of natural-polymer hydrogel packaging materials is not merely a breakthrough in materials science; their effectiveness must also be validated in complex food matrices.

For the specific spoilage mechanisms of different food systems, such as respiration, lipid oxidation, and microbial growth, hydrogel packaging has demonstrated highly targeted preservation efficacy [8,9]. The preservation performance of hydrogel-based packaging is commonly evaluated using total viable count (TVC), pH, total volatile basic nitrogen (TVB-N), thiobarbituric acid reactive substances (TBARS), color, firmness, weight loss, and sensory attributes. These parameters reflect microbial proliferation, protein and lipid degradation, moisture transfer, textural changes, and sensory-quality deterioration. In studies of aquatic products and meat, TVB-N is an integrated measure of volatile basic nitrogenous compounds generated during protein deterioration, whereas trimethylamine is one specific component of this group. Combining TVB-N with microbial counts, pH, TBARS, and sensory evaluation enables a more comprehensive assessment of packaging performance from microbiological, chemical, and sensory perspectives.

Because fruits and vegetables remain living tissues after harvest, the core of their preservation is to suppress respiration and reduce moisture transpiration. For easily damaged fruits and vegetables with irregular surfaces, extensible and self-sealing coatings are mainly constructed by dipping or spraying hydrogel solutions [10,11], simultaneously providing oxygen and moisture barriers, UV shielding, and sustained antimicrobial release, thereby effectively reducing respiration intensity and moisture loss. Reported preservation outcomes for fruits and vegetables vary with species, maturity, initial microbial load, coating thickness, gas exchange, relative humidity, temperature, and the endpoint used to define deterioration. Some systems also incorporate essential oils, nanoparticles, or antioxidants to achieve synergistic antimicrobial and antioxidant effects, making them suitable for highly perishable products such as berries, cut fruits, and leafy vegetables.

Aquatic products such as fish and shrimp have high moisture contents, near-neutral pH values, and abundant proteins. During storage and transportation, they continuously release water and acidic spoilage exudates, causing ordinary hydrophilic films to become rapidly wetted, dissolve, and fail. This high-humidity environment accelerates microbial proliferation and protein decomposition [12]. In addition, volatile basic nitrogenous compounds accumulate during storage, leading to increases in TVB-N values and, in many cases, pH. These processes are the fundamental causes of their perishability [10]; therefore, antimicrobial packaging is needed to inhibit microbial proliferation and extend freshness. First, edible or degradable carriers (such as anthocyanin films, superhydrophobic aerogels, and CA-DEA coatings) are used to establish liquid- and oxygen-barrier layers on high-moisture surfaces. Antimicrobial-antioxidant active ingredients (such as anthocyanins and citric acid) are then formulated as microcapsules or deep eutectic systems for sustained release, suppressing sharp increases in TVB-N/TBARS, while pH-based color development or exudate isolation is used to visualize freshness. In this way, In the studies considered here, changes in microbial counts, TVB-N, TBARS, pH, exudate accumulation, and sensory attributes were evaluated under different refrigerated-storage conditions [13,14,15].

Meat spoilage is primarily driven by microbial proliferation. Its metabolites (TVB-N and volatile amines) raise the pH, soften tissues, and produce off-odors [16], while endogenous proteases and lipid oxidation further accelerate protein degradation and flavor deterioration. One representative strategy for meat preservation is to construct a double-network sustained-release barrier, in which a rigid antimicrobial framework and a flexible oxygen-barrier network are crosslinked to form an interpenetrating double-network hydrogel film. This film can continuously release natural antimicrobial agents while blocking oxygen and ultraviolet radiation, significantly reducing TVB-N and total viable counts and delaying microbial and chemical deterioration under the refrigerated conditions reported in the individual studies [17,18].

According to the 2025 Bioplastics Market Development Update released by European Bioplastics (EUBP) on 2 December 2025, global biobased plastics production capacity is expected to double from 2.31 million metric tons in 2025 to approximately 4.69 million metric tons by 2030. Therefore, driven by the “dual-carbon” goals and plastic-restriction policies in various countries, the development of novel biobased, biodegradable, and intelligent packaging materials has become a research focus in materials science and food science [19,20]. However, despite the significant progress witnessed in recent years, the development of multifunctional hydrogel-based food packaging is confronted with a critical “trilemma”: the inherent conflict among robust mechanical strength, adequate moisture/oxygen barrier performance, and on-demand intelligent functionality [21]. Most existing reviews have primarily focused on summarizing material categories and preparation methods, yet they often fail to systematically deconstruct the underlying structure–property–function relationships that govern the practical translation of these materials [22,23,24]. Consequently, the field remains fragmented, with a noticeable gap between laboratory-scale innovations and industrially viable solutions [25,26,27].

**Figure 1:**
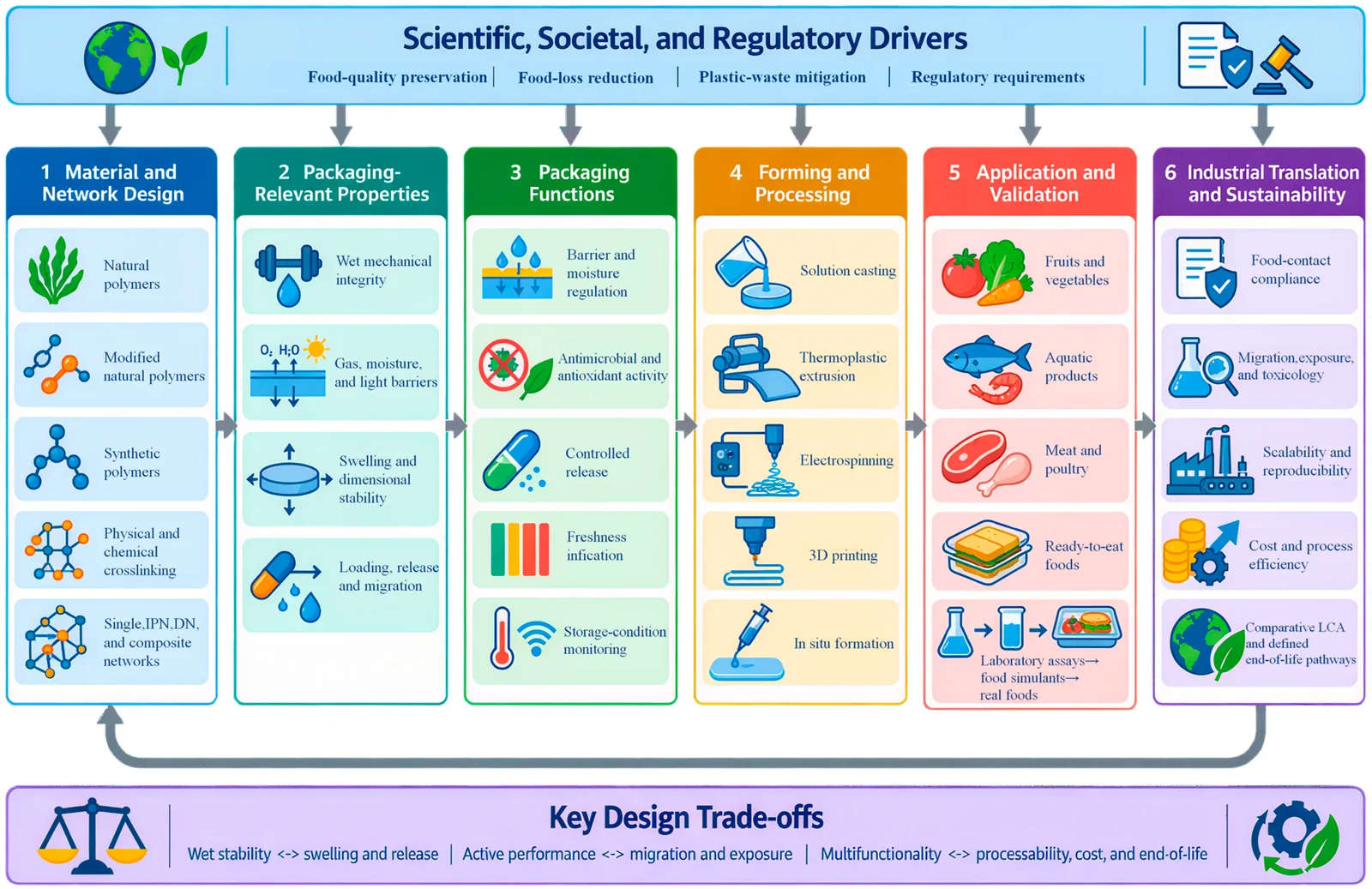
Integrated structure–property–function–processing–application–translation framework for hydrogel-based and hydrogel-derived food-packaging systems.

This review aims to transcend a conventional summary by offering a critical and integrated perspective that bridges materials science, food engineering, and sustainable manufacturing, as shown in Figure 1. We critically review recent advances in hydrogel-based packaging, yet our emphasis lies in elucidating the design logic—how molecular engineering (e.g., crosslinking strategies, nanocomposite reinforcement) and microstructure modulation (e.g., double-network, gradient structures) can synergistically resolve the abovementioned performance conflicts. Furthermore, we provide a comprehensive comparative analysis of forming and processing technologies—including solution casting, electrospinning, and 3D printing—with a focus on their scalability, economic viability, and compatibility with bio-based feedstocks. Importantly, we critically assess the bottlenecks that are rarely addressed: water sensitivity under high-humidity conditions, regulatory hurdles concerning active component migration, and the cost barriers hindering industrial adoption.

Web of Science Core Collection and Scopus were used as the primary databases to identify studies on hydrogel-based food packaging published between January 2015 and March 2026, with ScienceDirect and PubMed serving as supplementary sources. Earlier representative publications were also considered when they provided fundamental knowledge of hydrogel structures, established crosslinking mechanisms, or food-contact regulations. The literature search covered the material construction, performance regulation, active-agent delivery, quality-monitoring functions, and food-preservation applications of hydrogels, hydrogel-derived films and coatings, and hydrated nanofibrous networks. Particular attention was given to studies that clearly described material composition, network-formation mechanisms, processing methods, and packaging-relevant properties. Real-food storage studies, food-contact safety assessments, and relevant regulatory and standards documents were additionally considered to establish a body of evidence encompassing material fundamentals, functional performance, and application translation.

On this basis, the literature was analyzed using a structure–property–function–processing–application–translation framework. First, polymer composition, crosslinking strategy, and microscopic network architecture were related to mechanical performance, barrier behavior, swelling characteristics, active-agent loading and release, and intelligent-response functions. Second, the effects of different forming and processing technologies on material structure, functional retention, and manufacturing scalability were compared, while application effectiveness was evaluated using evidence from in vitro functional assays, food-simulant tests, and real-food storage studies. Finally, the requirements for engineering translation were examined in terms of migration and exposure, food-contact safety, regulatory compliance, economic feasibility, and full-life-cycle sustainability. Quantitative data were interpreted together with film thickness, temperature, relative humidity, testing method, and storage conditions to identify comparable performance trends and structure–function relationships among different systems. Compared with previous reviews [1,3,4,6] organized primarily by material category or individual function, this framework connects material design, functional performance, food validation, and industrial application, thereby providing a systematic basis for material selection, structural optimization, performance evaluation, and engineering development of multifunctional hydrogel-based packaging.

## 2. Polymer Substrates for Hydrogel-Based and Hydrogel-Derived Food-Packaging Materials

Hydrogel-based food-packaging materials can be constructed from polysaccharides, proteins, chemically modified biopolymers, water-soluble synthetic polymers, or combinations of these components. Polymer origin alone, however, does not adequately determine packaging performance. The formation and properties of these materials are jointly governed by molecular structure, functional-group chemistry, crosslinking mechanism, network density, moisture content, plasticization, and interactions among individual components. Accordingly, the materials discussed in this section are organized primarily according to their dominant network-forming components and structural roles rather than being placed within a rigid origin-based classification.

In this review, a hydrogel refers to a hydrated three-dimensional polymer network capable of retaining water, whereas a hydrogel-derived film refers to a dry or partially dried material obtained from a hydrogel-forming network or precursor. Hydrated nanofibrous membranes and interfacial coatings are included when they exhibit network formation, swelling, or water-mediated functional behavior. Conventional biopolymer films without a hydrogel-related network are considered only when they provide relevant evidence concerning material reinforcement, active-agent delivery, or packaging performance. Similarly, renewability, water solubility, disintegration, and mass loss are not treated as direct evidence of biodegradation or compostability. The environmental behavior of each material depends on its chemical composition, crosslinking density, additives, processing history, and disposal conditions. This structure-oriented approach enables polymer substrates to be evaluated in relation to network formation, packaging function, food-contact conditions, and end-of-life performance.

### 2.1. Natural-Polymer Substrates

Natural polysaccharides and proteins contain abundant hydroxyl, amino, carboxyl, and other reactive groups that facilitate hydration, intermolecular association, chemical crosslinking, and incorporation of active compounds. These characteristics make them suitable for constructing coatings, hydrogel films, hydrated nanofibrous membranes, and composite networks. Depending on the substrate category, they provide differentiated functions in food packaging systems, including barrier protection, antimicrobial activity, antioxidant activity, and intelligent detection.

**Figure 2:**
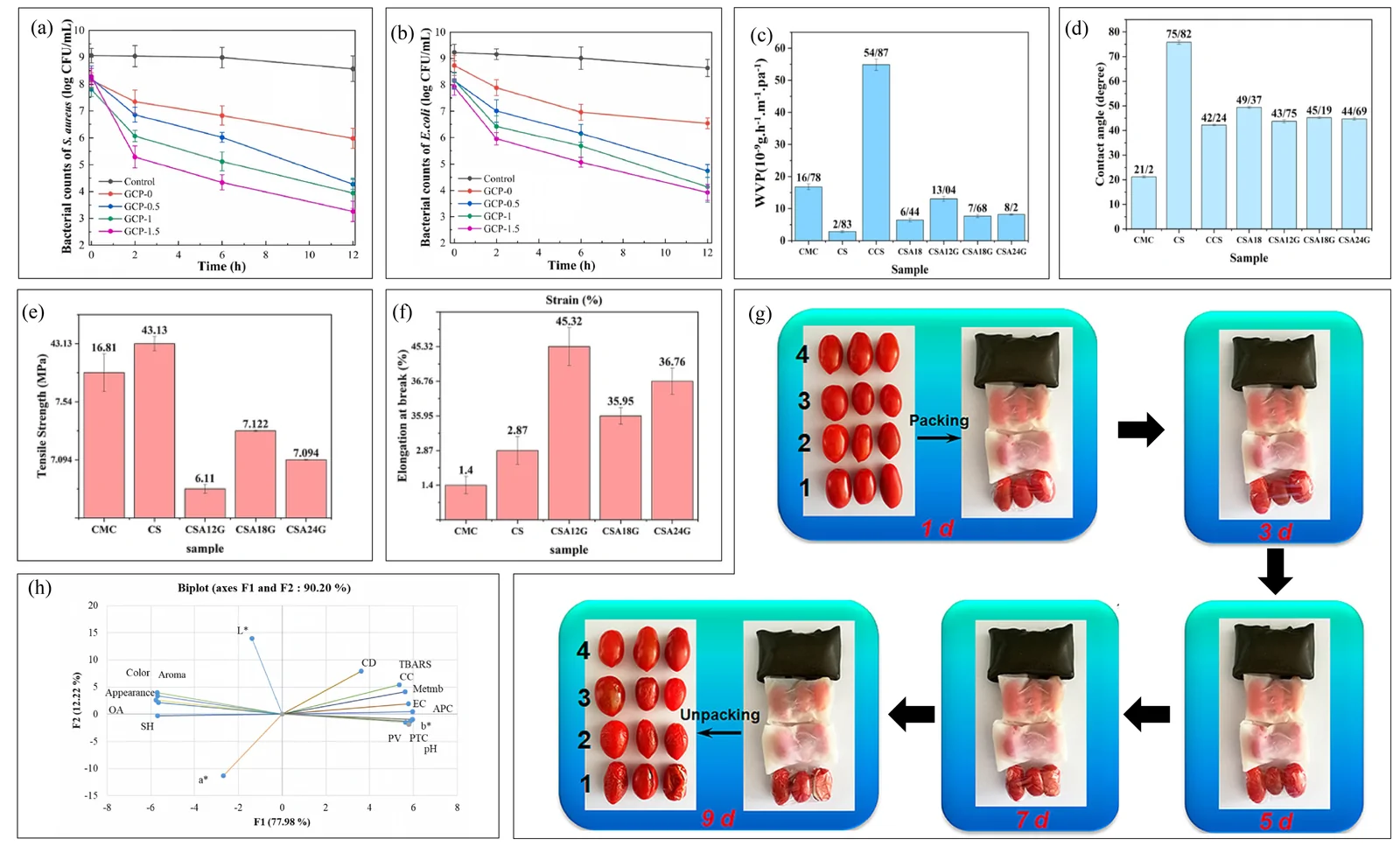
(**a**) Bacterial counts of *S. aureus* inactivated by hydrogels. (**b**) Bacterial counts of *E. coli* inactivated by hydrogels. Reproduced with permission [28]. Copyright 2022 Elsevier. (**c**) Water Vapor Permeability (WVP) of CSA18G. (**d**) Water contact angles on the surface of CSA18G. (**e**) Tensile strength of CSA18G. (**f**) Elongation at break of CSA18G. Reproduced with permission [29]. Copyright 2025 Elsevier. (**g**) Group 4 is Ag@HPAMAM NPs-embedded cellulose film. The packaging of cherry tomatoes can significantly extend the shelf life. Reproduced with permission [31]. Copyright 2020 AMER CHEMICAL SOC. (**h**) The impact of gelatin-NaAlg coating impregnated with BPE on physicochemical, microbial growth, color, and sensorial property values of meat samples over 14 days of storage. Adapted from Ref. [41] under the Creative Commons Attribution 4.0 International License (CC BY 4.0).

Polysaccharide-based hydrogels, owing to their good film-forming ability, strong hydrophilicity, and, in most cases, edibility, are widely used as coatings for fruit and vegetable preservation and as carriers for active substances. Chitosan is currently the most widely used active-packaging substrate. Because of its polycationic nature, chitosan hydrogels can directly disrupt cell membranes and show significant inhibitory effects against *Staphylococcus aureus*, *Escherichia coli*, and molds. Liu et al. [28] blended gelatin, chitosan, and 3-phenyllactic acid by electrospinning to prepare a nanofibrous membrane that formed a GCP-1 antimicrobial hydrogel after absorbing water. This hydrogel had a porous three-dimensional network structure, with significantly improved water absorption and stability. As shown in Figure 2a,b, experiments demonstrated that it effectively killed *S. aureus* and *E. coli* and extended the preservation period of refrigerated chicken breast to 4 days, indicating good biocompatibility and potential for food-packaging applications. Sabzevari et al. [29] used solution blending and thermal crosslinking to mix chitosan (CS) and carboxymethyl cellulose (CMC) at a 1:1 ratio, with citric acid (CA, 18 wt.%) as the crosslinker and glycerol as the plasticizer, thereby preparing a uniform and flexible CS-CMC hydrogel film (CSA18G). As shown in Figure 2c–f, the film exhibited low water vapor permeability (7.68 × 10^−9^ g·h^−1^·m^−1^·Pa^−1^), moderate hydrophilicity (contact angle of 45.19°), and good mechanical properties (tensile strength of 7.12 MPa and elongation at break of 35.95%). It effectively delayed broccoli spoilage at room temperature, demonstrating potential as a degradable food-packaging film. Godoy et al. [30] modified starch (m-St), isolated from the avocado seed and synthesized with tert-butyl acetoacetate (t-BAA), was embedded into polylactic acid (PLA) to design new eco-friendly composites. PLA/m-St (1:6) 20 wt% composites showed a dramatic increase in elongation at break (EB%) from 3.35 to 27.80% (about 730% enhancement) and exhibited remarkable UV-blocking performance from 16.21 to 83.86% for UVB, relative to pure PLA.

Cellulose and its derivatives (such as CMC and HPMC) are mainly used to construct high-strength packaging films. Nanocellulose (CNF/CNC) is often introduced into hydrogels as a reinforcing phase to form “bionanocomposites,” thereby addressing the low wet strength of natural hydrogels. Gu et al. [31] dissolved cotton fibers in an alkali/urea system, first oxidized cellulose with NaIO_4_ to generate aldehyde groups, and then used amino-terminated hyperbranched poly(amidoamine) for in situ reduction and anchoring of Ag nanoparticles. Ethanol regeneration yielded an Ag@HPAMAM-cellulose hydrogel. This hydrogel exhibited high antimicrobial activity, silver leakage below 10%, and simultaneously improved mechanical and barrier properties. When used for cherry tomato packaging, as shown in Figure 2g, it significantly extended shelf life. Jacob et al. [32] reviewed the preparation and packaging performance of PLA/nanocellulose biocomposites. These predominantly dry thermoplastic or solvent-cast composites are included here as hydrogel-related reference systems for evaluating the reinforcing and barrier contributions of nanocellulose rather than as hydrated hydrogels. Sarafidou et al. [33] used sugar beet pulp as a raw material, first extracting sugar liquor and fermenting it to synthesize bacterial cellulose (BC), and then preparing nanoscale BNC-A and BNC-E by sulfuric acid hydrolysis and enzymatic hydrolysis, respectively. These materials were blended with high-methoxyl pectin and cast into edible composite hydrogels. The resulting BNC-A hydrogel showed a 40% increase in tensile strength, a 54% increase in elongation, a hydrophobic surface with a contact angle of 107°, and shielding of more than 95% of UVA/UVB. It could delay light-induced lipid oxidation, discoloration, nutrient loss, and microbial growth, thereby extending shelf life and providing a new option for degradable, high-mechanical-strength active food-packaging films.

Protein-based hydrogels exhibit excellent oxygen-barrier properties, can be chemically modified and functionally designed, and are commonly used for packaging high-protein and high-fat foods. Gelatin films, in particular, have extremely low oxygen permeability and can effectively prevent oxidative discoloration of meat myoglobin and lipid oxidative rancidity. Zhou [34] encapsulated Angelica sinensis essential oil (AEO) in a gelatin solution by electrospinning to prepare gelatin/AEO nanofibrous hydrogel films with diameters of approximately 330–377 nm. The films possessed both antioxidant activity (maximum DPPH scavenging rate of 85%) and broad-spectrum antimicrobial activity (inhibition rates against *E. coli* and *S. aureus* increased with AEO concentration). The contact angle increased from 44.9° to 101.3°, significantly improving the water barrier. The MTT assay showed no marked cytotoxicity under the tested conditions; however, food-contact suitability would additionally require migration, exposure, residual-compound, and toxicological assessments. Castro et al. [35] used casting and oven drying to blend cassava starch with chicken-derived gelatin at different ratios and plasticized the blends with glycerol, producing edible composite films approximately 1.2 mm thick. Optimization using a simplex-lattice experimental design determined a starch-to-gelatin ratio of 53:47, giving the films moderate mechanical strength (Young’s modulus of approximately 200 MPa and elongation of approximately 28%) and relatively low water vapor permeability (0.41 g·mm·kPa^−1^·h^−1^·m^−2^). The formulated film had a smooth surface, good thermal stability, low water solubility and haze, and properties close to those of low-density polyethylene, allowing its direct use as degradable food packaging. Sooch et al. [36] synthesized Ti-doped CuO nanoparticles (10–13 nm) through gelatin-assisted in situ stabilization and coprecipitation, and uniformly dispersed them in a gelatin-glycerol matrix by solution casting to obtain a transparent and flexible nanocomposite film approximately 0.12 mm thick. Because the nanoparticles and gelatin formed a dense network, water vapor permeability decreased by 42%, tensile strength increased from 7.2 MPa to 15.3 MPa, and the contact angle increased to 95°, indicating enhanced hydrophobicity. The film produced significant inhibition zones against *E. coli*, *S. aureus*, and two fruit and vegetable pathogens, while exhibiting low cytotoxicity. When used to wrap cherry tomatoes, it preserved them for 18 days at 40 ± 3 °C, far longer than the 4 days achieved by ordinary films, and it completely degraded in soil within 9 days, thereby combining active function, degradability, and mechanical performance in food packaging.

In addition, plant proteins are abundant, inexpensive, and degradable. Structurally, they contain large numbers of polar amino acids that can form dense hydrogen-bonding networks, making oxygen difficult to penetrate while allowing water vapor to pass through, thereby suppressing the growth of harmful microorganisms and enabling active preservation [37]. Merino et al. [38] used hot-press molding to blend carrot pomace (rich in pectin-cellulose) with 50 wt.% wheat gluten and added 10 wt.% polyglycerol-3 as a plasticizer, producing transparent and flexible hydrogel films. The resulting films had an elastic modulus of 245 MPa, tensile strength of 10 MPa, and elongation at break of 25%, together with high transparency, effective UV shielding, antioxidant activity of 3.4 μmol TE/g, low water vapor permeability (1.3 × 10^−9^ g s^−1^ m^−1^ Pa^−1^), and low oxygen permeability (296 cm^3^ μm m^−2^ day^−1^ atm^−1^). They degraded by 83% in soil within 30 days, complied with EU migration limits for food-contact materials, and could be used directly as edible or compostable food packaging. Babayev et al. [39] used an antisolvent precipitation-pseudolatex technique to encapsulate carvacrol in zein nanocapsules (zNC), followed by casting at 50 °C to obtain transparent hydrogel films approximately 60 μm thick with 9% water content. The resulting films had stable mechanical properties, released approximately 50% of the carvacrol within 24 h, achieved 100% inhibition of *S. aureus*, and exhibited moisture absorption of no more than 3% and water solubility of no more than 10%. They therefore combined good water-barrier properties and degradability and were suitable for sustainable antimicrobial food packaging.

However, a single natural polymer often cannot simultaneously meet the stringent requirements of food packaging for mechanical strength, barrier properties, and functionality. Consequently, “polysaccharide-protein” and “polysaccharide-polysaccharide” composite hydrogels have become mainstream research directions. For example, positively charged chitosan can be combined with negatively charged gelatin through electrostatic interactions. The resulting hydrogel film not only integrates the antimicrobial properties of chitosan and the oxygen-barrier properties of gelatin, but also significantly improves the material’s water stability through the formation of a polyelectrolyte complex. Such biomimetically designed composite hydrogels provide a practical solution for achieving fully biodegradable, high-performance food packaging. Smaoui et al. [40] used seafood by-products (chitin and collagen) as raw materials to prepare chitosan-gelatin composite hydrogel films through solution casting, electrospinning, or hot-press molding. These composite hydrogel films combined high transparency, edibility, and degradability, and the incorporation of plant extracts or nanoparticles significantly improved their tensile strength, oxygen and moisture barrier properties, and antioxidant and antimicrobial performance. Such active packaging systems based partly on renewable feedstocks have shown potential for extending the shelf life of meat, fish, fruits, vegetables, and other foods, although the magnitude of preservation depends on the food matrix and storage conditions. Chaari et al. [41] used solution casting and oven drying to blend beet peel extract (BPE, 0.25–1%) with gelatin-sodium alginate, producing a red, flexible, edible composite film approximately 0.03 mm thick. Owing to polyphenol-polysaccharide hydrogen-bond crosslinking, the tensile strength increased from 14 MPa to 36 MPa, water swelling decreased by 36%, DPPH radical scavenging reached 70%, and clear inhibition zones were produced against foodborne bacteria. Vacuum-wrapping beef with the film and refrigerating it at 4 °C for 14 days significantly inhibited microorganisms and lipid/protein oxidation and maintained color and sensory scores, thereby markedly extending shelf life, as shown in Figure 2h. Elomar [42] used solution blow spinning to blend a gelatin-chitosan solution with 0.75 wt.% eucalyptus essential oil and prepare a nanofibrous hydrogel film. The resulting film had an average fiber diameter of approximately 260 nm, moderate air permeability, improved thermal stability, good antioxidant activity (76.9% DPPH scavenging), and antimicrobial activity (an inhibition zone of 11.3 mm against *S. aureus*). This biodegradable active film can simultaneously provide antimicrobial protection and environmental friendliness in food packaging and wound dressings. Representative quantitative properties and food-packaging application evidence for these materials are summarized in Table 1.

### 2.2. Synthetic/Semi-Synthetic Polymer Substrates

Although natural-polymer-based hydrogels offer the advantage of renewable resources, their low mechanical strength, inherent instability, and pronounced sensitivity to environmental humidity limit their large-scale use in industrial food packaging to some extent. Synthetic and semi-synthetic polymers, with controllable molecular structures, uniform film-forming properties, and excellent mechanical performance, provide an important supplement for constructing high-performance hydrogel packaging materials. In particular, polymers with good biocompatibility and biodegradability are gradually becoming a research focus.

**Figure 3:**
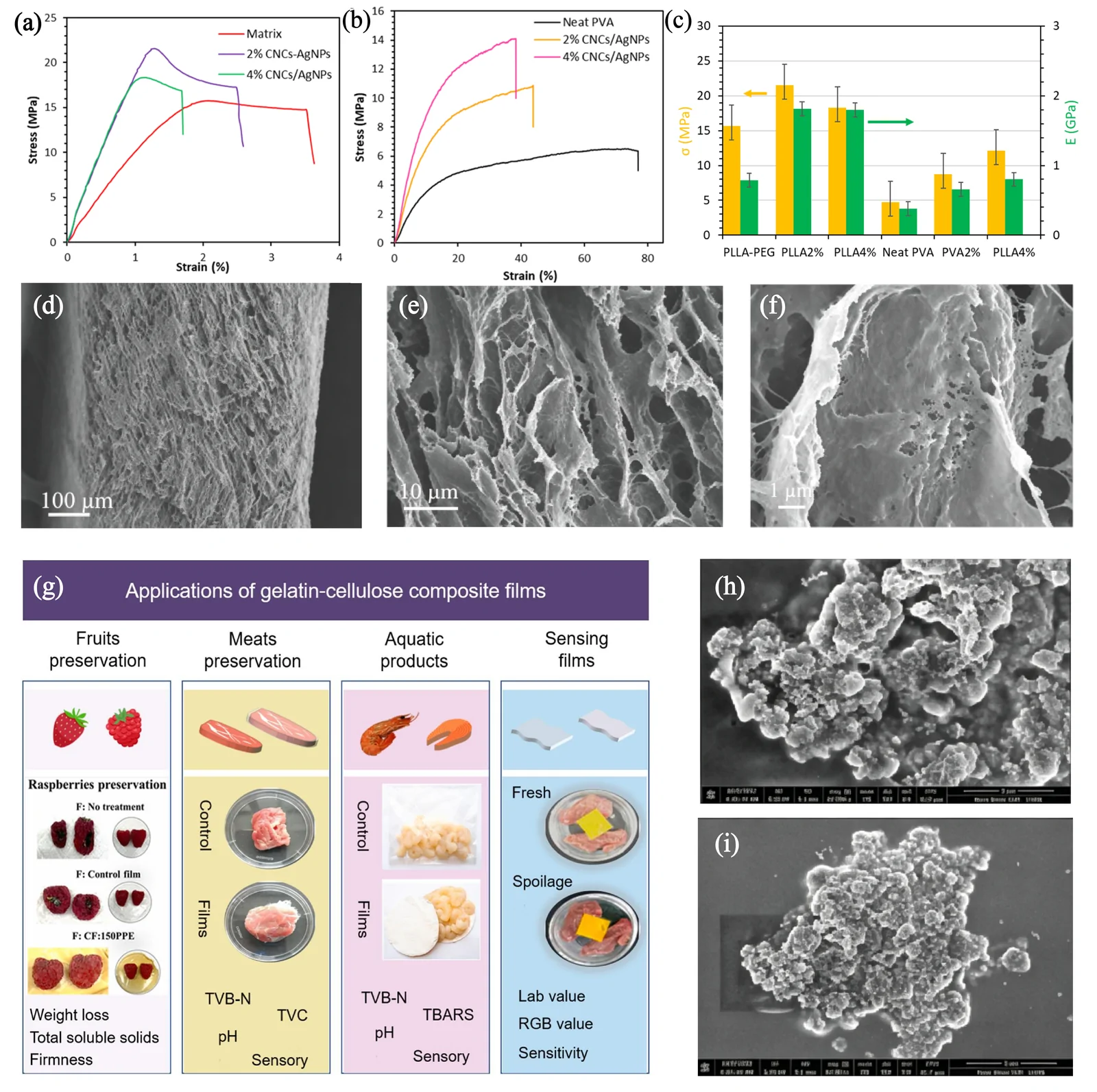
Stress–strain curves of PLLA-PEG (**a**) and PVA (**b**) films with different CNCs-AgNPs contents; (**c**) corresponding strength σ and modulus E. Reproduced from Ref. [49] with permission from the Royal Society of Chemistry. (**d**–**f**) Low- and high-magnification cross-sectional SEM images of CMC/PVA/PEI/TA_3_ hydrogel films. Reproduced with permission [11]. Copyright 2022 Elsevier. (**g**) Reported applications of gelatin–cellulose composite films in fruit, meat, and aquatic-product preservation and freshness indication. Reproduced with permission [51]. Copyright 2025 Elsevier. (**h**,**i**) Cross-sectional SEM images of SAlg/OkG films at a mass ratio of 85:15, recorded at ×2000 and ×5000 magnification. Reproduced with permission [52]. Copyright 2023 Elsevier.

Polyvinyl alcohol (PVA) is one of the most widely investigated synthetic polymers in hydrogel-based and hydrogel-derived packaging. Owing to its excellent water solubility and nontoxicity [11], strong film-forming ability [43], biocompatibility [44], high transparency [45], high oxygen-barrier performance [46], and tunable mechanical properties [47], PVA is a widely investigated film-forming polymer for hydrogel-derived and composite packaging systems. Ravindran et al. [48] used a 1:1 PVA:PEG blend film as the matrix and incorporated silver nanoparticles (AgNPs) green-synthesized using Capparis zeylanica leaf extract, producing composite films with uniform thickness, transparency, and enhanced thermal stability. The AgNPs imparted significant antimicrobial activity, producing clear inhibition zones against *E. coli*, *S. aureus*, and other microorganisms, while moderately increasing moisture absorption and reducing microbial degradation of the film. These results indicate potential for active food-packaging applications, although food-preservation effectiveness requires validation in real-food storage systems. The AgNPs-PEG/PVA nanocomposite film combined biodegradability with active-packaging functions, providing a safe and efficient solution for functional food-packaging applications. Aouay et al. [49] demonstrated the synergistic potential of periodate-oxidized cellulose nanocrystals (CNCs) decorated with silver nanoparticles (AgNPs) to enhance the antimicrobial and mechanical properties of poly(vinyl alcohol) (PVA) and poly(l-lactic acid) (PLLA) biopolymer matrices. The stress–strain plot for PVA and PLLA-PEG for neat films and at 2 and 4 wt% CNCs-AgNPs is presented in Figure 3a–c. At 2 and 4 wt% CNCs-AgNPs, the increment in σ reached 84 and 154%, respectively, while it was only 37 and 17% for the PLLA-PEG matrix. With the incorporation of 4 wt% CNCs-AgNPs, the modulus of PVA and PLLA films significantly increased by 25 and 75%, respectively, compared to their neat matrices. Zhao et al. [11] blended CMC, PVA, and TA at room temperature, then introduced PEI and allowed the reaction to proceed at 60 °C for 12 h. After freezing at −20 °C for 12 h and thawing at room temperature, they obtained a honeycomb-porous CMC/PVA/PEI/TA_3_ hydrogel film approximately 550 μm thick, whose SEM images are shown in Figure 3d–f. Multiple reversible noncovalent crosslinks, including hydrogen bonds, coordination bonds, and cation-π interactions, formed within the film, imparting comprehensive properties such as 400% elongation, room-temperature self-healing, 234 kPa adhesion, complete UV shielding, antioxidant activity, and low O_2_/water-vapor permeability. When strawberries, cherries, and mangoes were wrapped with this film and stored at room temperature for 7 days, they showed the lowest decay rate and best hardness retention, with shelf life extended by at least one week.

Semi-synthetic polymers are derivatives obtained by chemically modifying natural polymers. Such modification can overcome the poor solubility, processing difficulty, and low stability of natural polymers while retaining their biodegradability and biocompatibility, thereby yielding packaging materials that combine high mechanical strength, excellent water and oxygen barrier properties, antimicrobial and antioxidant activities, biodegradability, and intelligent color indication [50]. Ding et al. [51] treated cellulose (CMC, HPMC, CA, EC, CNC, and CNF) through chemical modifications such as carboxymethylation, hydroxypropylation, and acetylation, or through nanosizing, and then blended and crosslinked it with gelatin. This overcame the low mechanical strength, poor water and oxygen barrier properties, and insufficient thermal stability of pure gelatin films, producing composite films with high mechanical performance, excellent barriers, and antimicrobial and antioxidant functions. As shown in Figure 3g, these films can replace petroleum-based plastics for long-term preservation and visual freshness monitoring of fruits, meat, and aquatic products. Saleem et al. [52] combined sodium alginate (SAlg) and okra gum (OkG) through physical blending and calcium-ion crosslinking to form an interpenetrating-network hydrogel film. SEM images of the 85:15 SAlg/OkG ratio are shown in Figure 3h,i. This strategy overcame the poor thermal stability, low mechanical strength, and excessive hydrophilicity of pure SAlg films while providing antioxidant activity and controllable degradation, allowing safe use in food packaging.

Food-contact suitability requires assessment of the finished formulation, including migration, exposure, residual monomers and crosslinkers, reaction by-products, degradation products, and the intended conditions of use. Biocompatibility or prior food use of an individual constituent does not independently establish compliance of the finished material.

**Table 1:** Representative quantitative properties and application evidence for selected hydrogel-based, hydrogel-derived, and related biopolymer food-packaging materials. (Note: Quantitative values are representative results reported for the indicated formulations and are not directly comparable across studies because of differences in composition, film thickness, hydration state, testing methods, relative humidity, temperature, and specimen geometry).

Representative Formulation	Structure or Processing	Representative Quantitative Properties	Functional or Application Evidence	Ref.
CS/CMC, 1:1, crosslinked with 18 wt.% CA and plasticized with glycerol	Thermally crosslinked hydrogel-derived film	TS 7.12 MPa; EAB 35.95%; Young’s modulus 0.22 MPa; WVP 7.68 × 10^−9^ g·h^−1^·m^−1^·Pa^−1^; WCA 45.19°	Material characterization; broccoli preservation evaluated at 21 ± 1 °C and approximately 33% RH for 7 days.	[29]
PLA containing 20 wt.% modified avocado-seed starch	Solvent-processed PLA/starch reference composite	EAB increased from 3.35% to 27.80%; UVB shielding increased from 16.21% to 83.86%	Material-level mechanical and optical evidence; not a hydrated hydrogel	[30]
Pectin/BNC-A composite	Cast biopolymer network	TS increased by 40%; EAB increased by 54%; WCA 107°; UVA/UVB shielding >95%	Material-level performance; food-preservation effectiveness requires product-specific validation	[33]
Cassava starch/chicken gelatin, 53:47	Cast and oven-dried film, approximately 1.2 mm thick	Young’s modulus ~200 MPa; EAB ~28%; WVP 0.41 g·mm·kPa^−1^·h^−1^·m^−2^	Material-level evidence; “direct use” and biodegradation claims require separate food-contact and end-of-life evidence	[35]
Gelatin/Ti-doped CuO	Cast nanocomposite film, approximately 0.122 mm thick	TS 7.2 → 15.3 MPa; WVP decreased by ~42%; WCA 95°	In vitro inhibition zones; tomatoes maintained for up to 18 days at 40 ± 3 °C versus pronounced deterioration of controls after ~4 days	[36]
Carrot pomace/50 wt.% wheat gluten/10 wt.% polyglycerol	Hot-pressed biocomposite	TS 10.1 ± 0.7 MPa; modulus 244.9 ± 25.3 MPa; EAB 24.7 ± 5.7%; WVP 1.3 × 10^−9^ g s^−1^ m^−1^ Pa^−1^; OP 296.1 ± 1 cm^3^ μm m^−2^ day^−1^ atm^−1^; overall migration 6.7 ± 0.6 mg dm^−2^	Antioxidant capacity 3.4 μmol Trolox g^−1^; 83% degradation reported after 30 days in soil under the authors’ test conditions	[38]
Zein nanocapsule film containing carvacrol	Pseudolatex casting at 50 °C; thickness ~60 μm	~50% carvacrol release in 24 h; moisture absorption ≤ 3%; water solubility ≤ 10%	Growth of *S. aureus* blocked under dynamic in vitro conditions; no real-food test	[39]
Gelatin/NaAlg containing 1% BPE	Solution-cast film, thickness ~0.03 mm	TS ~14 → 36 MPa; swelling decreased by 36%; DPPH scavenging ~70%	In vitro antimicrobial and antioxidant assays; minced beef evaluated for 14 days at 4 °C under vacuum packaging	[41]
CMC/PVA/PEI/TA_3_	Freeze-thaw, multi-interaction hydrogel film; thickness ~550 μm	EAB ~400%; adhesion 234.08 kPa; oxygen permeability 32.64 cm^3^·μm m^2^·day·kPa; WVTR 642.92 g/m^2^·24 h	Strawberries, cherries, and mangoes evaluated under ambient conditions for 7 days	[11]
NCFs/SA/CaCl_2_-5	Freeze-thaw and Ca^2+^ ionic crosslinking	EAB ~900%; transparency ~90%; conductivity 2.34 S/cm	Beef preservation evaluated at 37 °C; exact control endpoint should be verified before stating the magnitude of shelf-life extension	[53]
SPI/DAS/15TA	Schiff-base and hydrogen-bonded film	TS increased by 208% relative to pure SPI; UV absorption edge 389 nm	*S. aureus* inhibition demonstrated in a laboratory assay; no real-food validation reported	[54]
SA-CFP double network	Evaporation-induced assembly followed by lanthanide-ion crosslinking	TS 43 MPa; transparency 89.6%; wet mechanical response reported after immersion	Biogenic-amine-responsive fluorescence; food-specific calibration remains necessary	[55]

## 3. Network-Formation and Crosslinking Strategies for Hydrogel-Based Packaging Materials

The three-dimensional network structure of hydrogel-based materials is established through physical interactions, chemical covalent bonds, or combinations of both. Network construction controls not only mechanical strength, swelling, thermal stability, and barrier performance but also active-agent retention, release, and potential migration. It also plays a decisive role in the environmental breakdown of the resulting material by regulating water penetration, polymer-chain mobility, and the accessibility of hydrolytic agents, enzymes, and microorganisms to susceptible chemical bonds. Reversible physical junctions may facilitate swelling, dissolution, or disintegration, whereas a high density of stable covalent crosslinks can restrict molecular diffusion and retard polymer-chain cleavage. Conversely, hydrolysable or enzyme-cleavable crosslinking units may promote degradation under appropriate environmental conditions. These processes are governed by the complete formulation—including the polymer chemistry, crosslinking mechanism and density, plasticizers, active compounds, and reinforcing fillers—and should not be interpreted as evidence of biodegradation unless biological conversion has been demonstrated under defined conditions. For food-contact applications, the choice of crosslinker must therefore be evaluated together with reaction conversion, residual content, possible reaction by-products, network stability, degradation products, and the intended conditions of use.

### 3.1. Physical Crosslinking

Physical crosslinking mainly relies on physical entanglement or secondary-bond interactions between molecular chains. This process generally requires no exogenous chemical reagents, proceeds under mild reaction conditions, and often forms reversible gel networks, which facilitates material processing and recycling.

**Figure 4:**
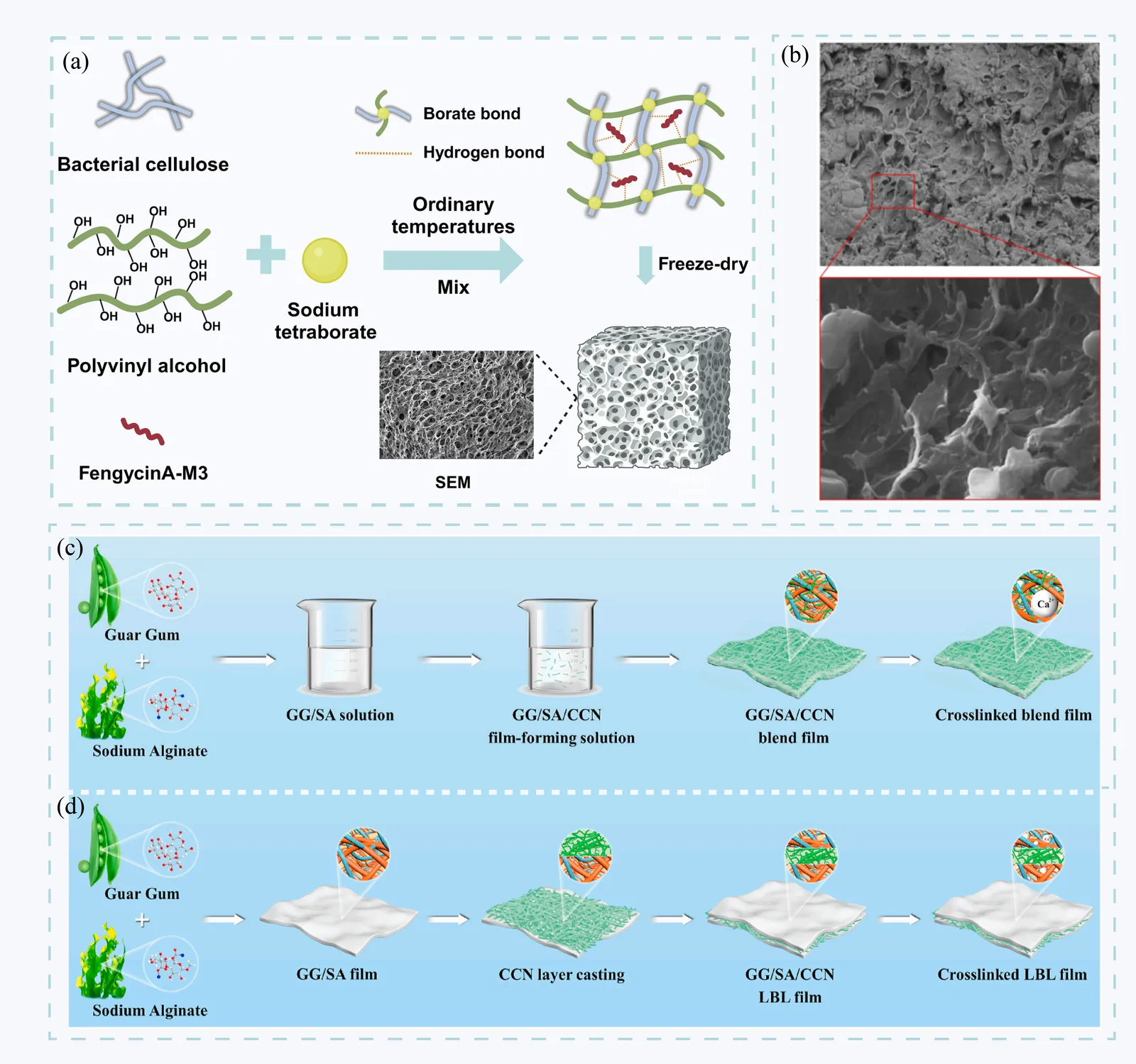
Physical-network construction in alginate-based composite hydrogels and films. (**a**) Preparation of an NCF/SA/CaCl_2_ composite hydrogel by freeze-thaw treatment and Ca^2+^ crosslinking. (**b**) SEM images of the NCF/SA/CaCl_2_-5 network at ×10,000 and ×30,000 magnification. Reproduced with permission [53]. Copyright 2024 Elsevier. (**c**,**d**) Preparation of GG/SA/CCN films by direct blending and layer-by-layer assembly followed by Ca^2+^ crosslinking. Reproduced with permission [59]. Copyright 2024 Elsevier.

Hydrogen bonding is the most common physical crosslinking mechanism in natural polymers. Polysaccharide and protein chains contain numerous -OH, -NH_2_, and -COOH groups, and during hydrogel formation these polar groups form dense intramolecular or intermolecular hydrogen bonds, as shown in Figure 4a [56]. Sana et al. [57] reviewed recent advances in locust bean gum (LBG) polysaccharides for sustainable food packaging and drug delivery, emphasizing their film-forming, gelling, and sustained-release properties. LBG is rich in hydroxyl groups and can form hydrogen-bonding networks with water, active components, or other polymers, thereby imparting mechanical strength, barrier properties, and controlled-release functions. This is the core mechanism underlying its use as a natural carrier and in intelligent packaging. However, hydrogen bonds are sensitive to water and temperature; therefore, hydrogels crosslinked solely through hydrogen bonding have poor water resistance and usually need to be combined with other crosslinking modes.

Electrostatic interactions use the attraction between oppositely charged polymers or ions to form physical crosslinking points. This mechanism is particularly common in polyelectrolyte hydrogels. For example, Ca^2+^ undergoes ion exchange with negatively charged carboxylate groups on sodium alginate chains and establishes electrostatic crosslinks, forming a dense network that both immobilizes hydrophilic groups to reduce moisture-induced plasticization and exposes more hydrophobic regions, thereby maintaining an effective oxygen barrier even under high-humidity conditions [58]. As shown in Figure 4b, Yu et al. [53] used soybean-husk nanocellulose fibers (NCFs) as a reinforcing phase, blended them with sodium alginate (SA), subjected the mixture to repeated freeze-thaw cycles at −80 °C, and performed in situ ionic crosslinking in a 3% CaCl_2_ solution. The resulting composite hydrogel combined high elongation (~900%), high transparency (~90%), and high conductivity (2.34 S cm^−1^). When used to preserve beef at 37 °C, the film extended shelf life from 2 days to 5 days, providing a new approach to intelligent and degradable meat packaging. As shown in Figure 4c,d, Liu et al. [59] first blended guar gum (GG) with sodium alginate (SA) to form films, then introduced carboxylated cellulose nanofibers (CCN) using either a blending method or a layer-by-layer self-assembly (LBL) method, and finally crosslinked the films with Ca^2+^ ions. This systematically improved the films’ mechanical properties, water barrier, light barrier, and water resistance, yielding biobased composite films suitable for high-moisture food packaging.

### 3.2. Chemical Crosslinking

To improve the mechanical strength and environmental stability of physically crosslinked hydrogels, chemical crosslinking locks the three-dimensional network by introducing stable covalent bonds between molecular chains. In food packaging, research focuses on chemical reactions that can proceed under relatively mild conditions; however, conversion, residual reagents, catalysts, and reaction by-products must be evaluated for the finished food-contact material.

**Figure 5:**
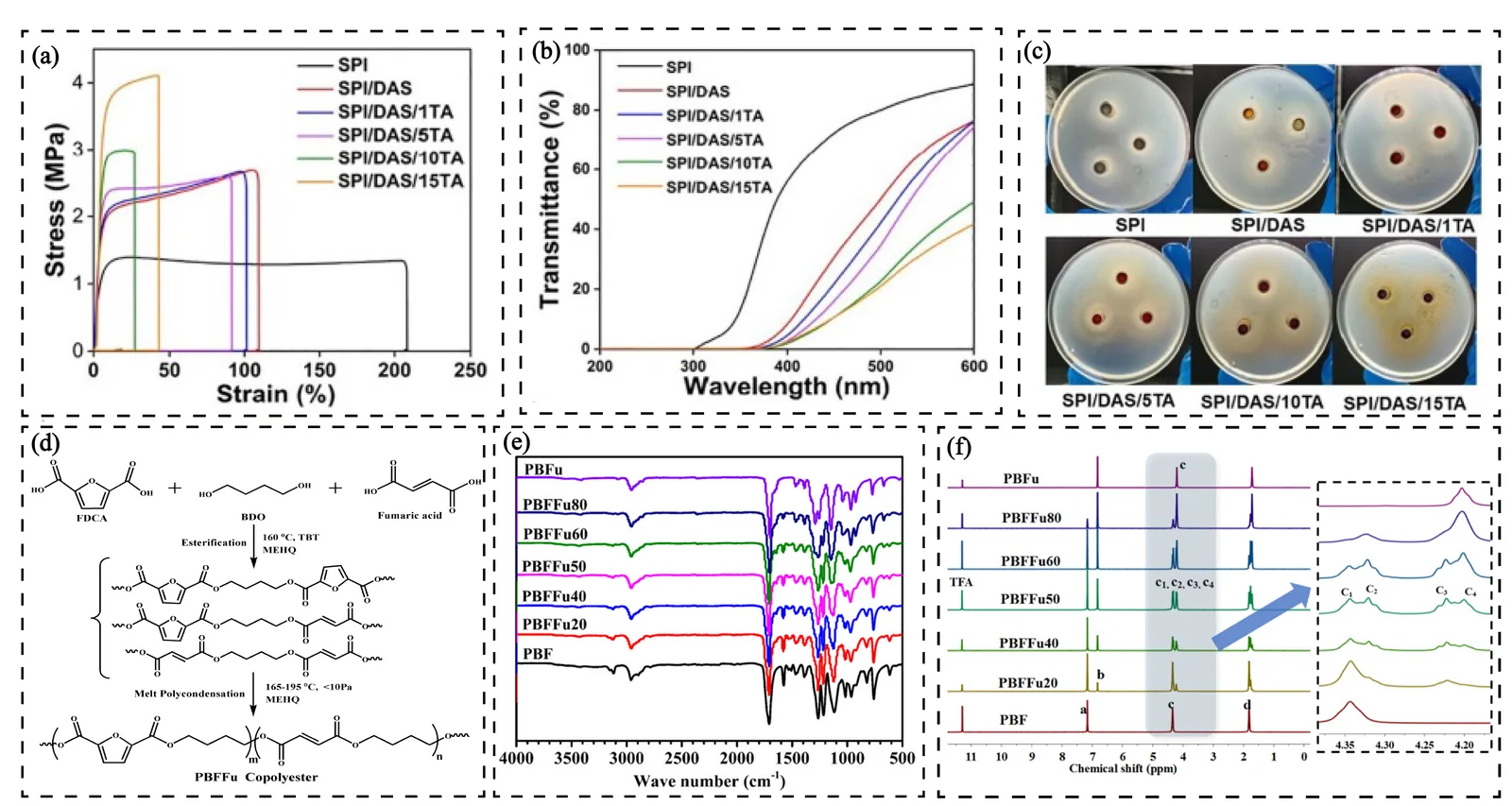
(**a**) Mechanical properties of SPI/DAS/15TA. (**b**) Transmittance of SPI/DAS/15TA. (**c**) Antibacterial activity of the films against the bacterial pathogen *S. aureus.* Reproduced with permission [54]. Copyright 2024 ELSEVIER SCI LTD. (**d**) Synthesis of PBFFu Copolyesters from FDCA, BDO, and FA. (**e**) FTIR spectra of PBFFu. (**f**) ^1^H NMR spectra for PBF, PBFu, and PBFFu. Reproduced with permission [64]. Copyright 2023 AMER CHEMICAL SOC.

Among these reactions, the Schiff-base reaction is a condensation reaction between aldehyde groups (-CHO) and amino groups (-NH_2_) that generates imine bonds (-C=N-). This Schiff-base-forming process can construct a three-dimensional network [60], thereby substantially improving the mechanical properties, water resistance, thermal stability, and barrier properties of films [61,62]. Zhao et al. [54] used soy protein isolate (SPI) as the base material and introduced dialdehyde starch (DAS) and different concentrations of tannic acid (TA), blending them under alkaline conditions to form films and systematically investigating the effects of TA on film structure and properties. As shown in Figure 5a–c, when the TA content reached 15 wt.%, the tensile strength of the SPI/DAS/15TA film increased by 208% relative to the pure SPI film, the ultraviolet absorption edge extended to 389 nm, and *S. aureus* was significantly inhibited, demonstrating potential for food-packaging applications.

Another common chemical crosslinking reaction is the Michael addition, a nucleophilic conjugate-addition reaction that generally involves a nucleophile (Michael donor, such as a thiol or amine) and an activated alkene (Michael acceptor, such as an acrylate or maleimide) [54,63]. Luan et al. [64] introduced biobased fumaric acid (FA) into poly(butylene succinate-co-butylene 2,5-furandicarboxylate) copolyesters (PBFFu) through melt polycondensation. Characterization of PBFFu is shown in Figure 5d–f. The resulting fully biobased material combined high mechanical strength, excellent water and oxygen barrier properties, and room-temperature post-functionalizability. Subsequently, the fumarate double bonds in the PBFFu main chain were used to graft side groups through thiol-Michael addition with β-aminothiol at room temperature, positioning the side-chain amino groups adjacent to ester bonds. Upon heating, intramolecular aminolysis promoted main-chain cleavage and molecular-weight reduction, thereby enabling thermally triggered degradation rather than improving structural stability.

## 4. Functional Design Strategies for Food Packaging

Although hydrogels possess excellent biological properties, their high water content results in a low Young’s modulus, while their high brittleness in the dry state often prevents them from meeting the tensile, puncture-resistance, and moisture-resistance requirements of food packaging during transportation and stacking. To overcome this bottleneck, researchers have developed various mechanical reinforcement strategies based on microstructural design principles. These strategies aim to improve comprehensive mechanical performance by strengthening the network skeleton, introducing energy-dissipation mechanisms, and constructing hierarchical structures.

### 4.1. Strategies for Enhancing Mechanical Properties

**Figure 6:**
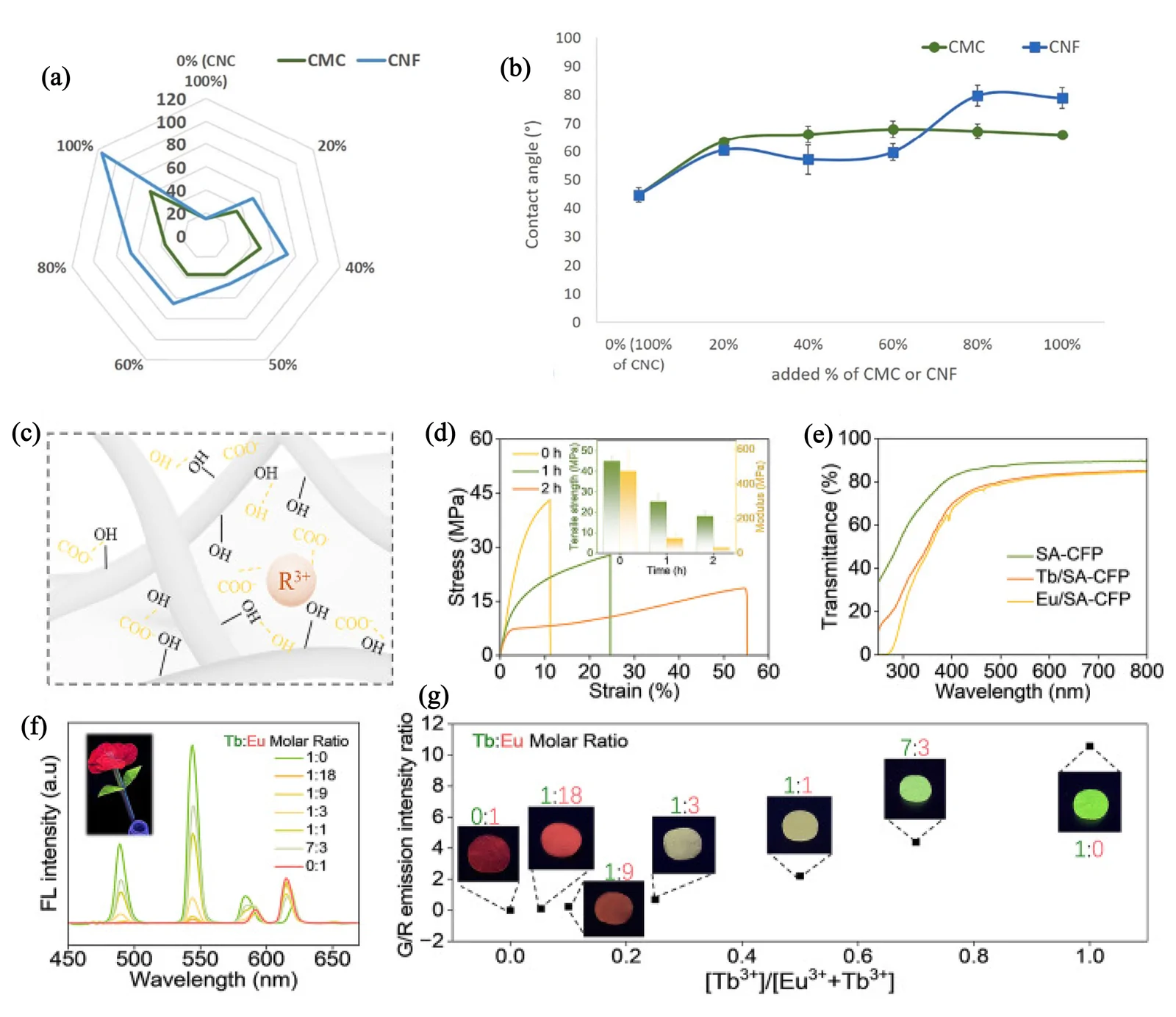
Effects of nanocellulose reinforcement and double-network construction on mechanical, barrier, and optical-response properties. (**a**) Tensile strength and (**b**) water-vapor transport performance of CNC-CMC and CNC-CNF films with different blend ratios. Reproduced with permission [68]. Copyright 2022 Elsevier. (**c**) The schematic diagram of interaction of SA-CFP film with lanthanide ions. (**d**) The wet tensile stress-strain curves and Young’s modulus of the SA-CFP composite film immersed in water for various times. (**e**) UV–vis transmission rate of the SA-CFP film and R/SA-CFP (R = Eu^3+^/Tb^3+^). (**f**) Fluorescence spectra (λex = 393 nm) of multicolor fluorescent film, which is prepared by immersing the SA-CFP film into mixed solutions of Eu^3+^ and Tb^3+^. (**g**) The green/red (G/R) emission intensity ratio as a function of the Tb^3+^/Eu^3+^ molar ratio (0:1,1:18, 1:9, 1:3, 1:1, 7:3, 1:0) and the corresponding photographs. Scale bar is 1 cm. All photos were taken under a 254 nm UV lamp. Reproduced with permission [55]. Copyright 2025 ELSEVIER SCI LTD.

The incorporation of nanofillers is the most direct and effective method for improving the mechanical strength of hydrogels. Cellulose nanofibrils (CNF), owing to their high aspect ratio and the tendency of fibers to become entangled, can provide good toughness and impact resistance when forming network structures [65]. When CNF is used in composite materials, it can effectively disperse stress and improve material flexibility. Cellulose nanocrystals (CNC), because of their inherently rigid structure, are more effective in strengthening materials [66]. Adding CNC to composite materials can significantly increase tensile strength and modulus, although toughness may decrease [67]. Fernandez-Santos et al. [68] blended CNC with either CNF or carboxymethyl cellulose (CMC) at different ratios and cast the mixtures into films, systematically comparing their mechanical, barrier, and degradation properties. As shown in Figure 6a,b, 40% CNF increased tensile strength by approximately fourfold, while more than 60% CMC still significantly reduced water vapor permeability at high humidity (90% RH). In practical olive preservation, CNC-CMC films outperformed plastic and aluminum foil.

In addition, single-network hydrogels often cannot simultaneously provide both rigidity and toughness. Interpenetrating polymer network (IPN) and double-network (DN) technologies entangle two polymer networks with complementary properties at the molecular scale and transfer stress through interfacial hydrogen bonding or covalent interactions, achieving a synergistic effect greater than the sum of the individual components [69]. Chen et al. [55] prepared sodium alginate-cellulose fiber paper (SA-CFP) double-network composite films through evaporation-induced self-assembly, achieving high transparency (89.6%) and high tensile strength (43 MPa). As shown in Figure 6c–g, subsequent crosslinking with lanthanide ions endowed the films with excellent wet strength and tunable fluorescence, enabling a rapid response to biogenic amines for intelligent food packaging.

A cross-study comparison demonstrates that network densification and reinforcement can improve mechanical and barrier properties through different mechanisms, although the reported values are not directly interchangeable because film thickness, hydration state, specimen geometry, relative humidity, and testing protocols differ among studies. The citric-acid-crosslinked CS/CMC film containing 18 wt.% citric acid exhibited a tensile strength of 7.12 MPa, an elongation at break of 35.95%, and a water-vapor permeability of 7.68 × 10^−9^ g·h^−1^·m^−1^·Pa^−1^ [29]. The carrot-pomace/wheat-gluten film plasticized with 10 wt.% polyglycerol showed a tensile strength of 10.1 ± 0.7 MPa, an elastic modulus of 244.9 ± 25.3 MPa, an elongation at break of 24.7 ± 5.7%, a water-vapor permeability of 1.3 × 10^−9^ gs^−1^ m^−1^ Pa^−1^, and an oxygen permeability of 296.1 ± 1 cm^3^ μm m^−2^ day^−1^ atm^−1^ [38]. By comparison, incorporation of Ti-doped CuO nanoparticles increased the tensile strength of a gelatin film from 7.2 to 15.3 MPa and decreased its water-vapor permeability by approximately 42% [36], while incorporation of beetroot-peel extract into a gelatin-sodium alginate film increased tensile strength from approximately 14 to 36 MPa and reduced swelling by 36% [41]. The SA-CFP double-network film reached a tensile strength of 43 MPa [55], whereas the hydrated NCFs/SA/CaCl_2_ network and CMC/PVA/PEI/TA_3_ network achieved elongations of approximately 900% and 400%, respectively [11,53]. These results indicate that dense covalent or nanofiller-reinforced networks generally favor strength and barrier performance, whereas hydrated reversible networks can provide substantially greater extensibility. Direct ranking should nevertheless be restricted to materials tested under comparable hydration and environmental conditions.

### 4.2. Active-Packaging Functions

As consumer requirements for food freshness and safety continue to rise, conventional materials that provide only physical barrier functions can no longer meet demand. Active-packaging technology has therefore emerged, with the core concept of establishing dynamic interactions among the packaging system, the food, and the internal environment. By loading functional active agents into hydrogels or biopolymer matrices, packaging materials can be endowed with antimicrobial, antioxidant, and environmental-regulation capabilities, thereby actively suppressing spoilage and enabling a transition from simple containers to active preservation systems.

**Figure 7:**
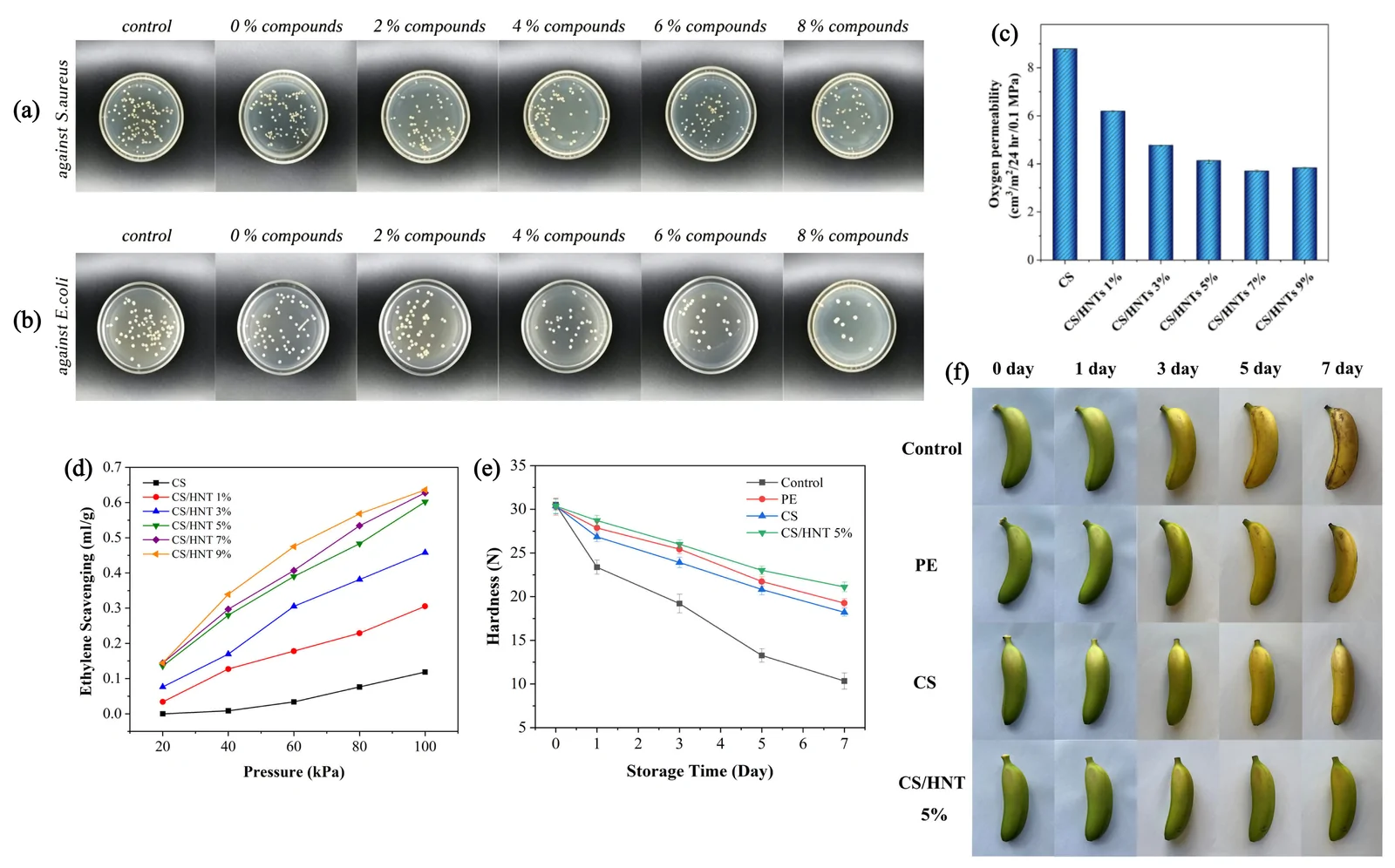
Antimicrobial, barrier, ethylene-scavenging, and food-application performance of active packaging films. (**a**,**b**) Laboratory antibacterial efficacy of CA-HNT/zein films against *S. aureus* and *E. coli*. Reproduced with permission [74]. Copyright 2022 Elsevier. (**c**) Oxygen permeability and (**d**) ethylene adsorption isotherms of chitosan films containing different HNT contents. (**e**) firmness of cherry tomatoes packaged with PE, chitosan, or CS/HNT 5% films and of unpackaged controls; and (**f**) appearance of similarly packaged bananas during storage. Reproduced with permission [84]. Copyright 2022 Wiley.

Microbial contamination is a major cause of food spoilage. Because of their unique three-dimensional network structure, hydrogel substrates can serve as excellent carriers for various antimicrobial agents, thereby constructing efficient antimicrobial systems. The most common approach is to create a sustained and controllable antimicrobial atmosphere within the packaging system, thereby inhibiting microorganisms on the food surface and in the packaging environment [70]. This process can be divided into three steps. First, carriers such as microcapsules, nanoemulsions, Pickering emulsions, multilayer films, electrospun nanofibers, halloysite nanotubes, sachets, or absorbent pads are used to encapsulate volatile and oxidation-sensitive essential oils, preventing burst release and loss during processing or early storage. Second, the carrier’s diffusion barrier, layer-by-layer electrostatic interactions, solid-particle interfaces, temperature/humidity response, or concentration gradients are used to slowly release the essential oils over time into the packaging headspace or onto the food surface, thereby maintaining a sufficient antimicrobial concentration. Finally, the released essential-oil vapor (or nanoparticles/phages) directly contacts microorganisms and disrupts membrane structures, enzyme systems, or genetic material, suppressing the growth of bacteria, yeasts, and molds and thereby extending shelf life [71,72,73]. Zheng et al. [74] loaded volatile carvacrol (CA) into halloysite nanotubes (HNTs) using a vacuum-acidification method, achieving a high loading of 17% and significantly improving thermal stability. After CA-HNTs were dispersed in a zein film, as shown in Figure 7a and Figure 7b, inhibition rates against *E. coli* and *S. aureus* reached 85% and 59%, respectively, and the storage life of cherry tomatoes at 23 °C was extended to 15 days. In addition, to prevent antimicrobial agents from migrating into food and altering its original odor, antimicrobial components can be immobilized on polymer chains through chemical bonds. The inherent positive charge of chitosan (CS) enables strong electrostatic interactions with negatively charged bacterial cell membranes, forming electrostatically mediated membrane disruption in situ on the bacterial membrane surface and significantly improving emulsion structural stability and the sustained-release performance of antimicrobial active substances. This positive-charge-mediated interfacial self-assembly strategy not only gives films long-lasting antimicrobial activity, but also extends shelf life [75]. In situ loading of metal nanoparticles (Ag and ZnO) in natural-polymer matrices gives films the ability to continuously release metal ions, disrupt bacterial membrane structures, and inhibit bacterial metabolism. At the same time, immobilizing the nanoparticles with carriers/crosslinkers such as hyperbranched polymers or polyphenols both reduces the risk of metal leakage and improves antimicrobial efficiency and the mechanical and barrier properties of the films [31,76]. Immobilization may reduce nanoparticle or metal-ion release, but food-contact suitability still requires particle- and ion-specific migration, exposure, and toxicological assessment. For example, Li et al. [77] generated silver nanoparticles (AgNPs) in pectin/gelatin films through in situ reduction of silver nitrate. The continuously released Ag^+^ disrupted bacterial membrane proteins and DNA, achieving nearly 100% inhibition of *E. coli* and *S. aureus*. AgNPs and curcumin acted synergistically against bacteria, while curcumin also served as a reducing agent during nanosilver formation, ensuring uniform AgNP dispersion and reducing the required dosage, thereby combining efficient antimicrobial action with biosafety. Sun et al. [78] uniformly embedded approximately 30-nm ZnO nanoparticles in a konjac glucomannan/chitosan matrix. The continuous release of Zn^2+^ disrupted bacterial membrane structures and produced clear inhibition zones against *E. coli* and *S. aureus*, yielding efficient antimicrobial activity. By filling micropores in the film and strengthening intermolecular interactions, the ZnO nanoparticles significantly improved film antimicrobial activity at a low loading and compensated for the weak intrinsic antimicrobial activity of mulberry anthocyanins.

Rancidity caused by lipid oxidation and enzymatic browning are major causes of quality deterioration in meat, nuts, and fresh-cut fruits and vegetables. Antioxidant packaging protects food by scavenging free radicals in the system or interrupting oxidation chain reactions. Oxidative reactions are among the principal causes of food quality deterioration: lipids, proteins, pigments, and other components are attacked by reactive oxygen species (ROS), which destroy nutrients such as essential fatty acids, proteins, and vitamins and generate aldehydes, ketones, acids, off-odors, discoloration, and reduced nutritional value [79]. In addition, microbial contamination and environmental factors (such as humidity, oxygen, and light) can accelerate food deterioration. Antioxidant packaging incorporates natural antioxidants (such as vitamin E, tea polyphenols, and rosemary extract) into packaging materials and uses controlled-release mechanisms to slowly migrate the antioxidant components to the food surface or interior, thereby continuously scavenging free radicals and inhibiting lipid oxidation. This method not only reduces the amount of antioxidants required for direct addition, but also forms a protective barrier on the food surface, delays oxidation, and extends food shelf life [80]. Zanela et al. [81] directly blended leaf powder from the Brazilian native plant Baccharis dracunculifolia, used as a natural antioxidant, into blown cassava starch/poly(butylene adipate-co-terephthalate) (PBAT) films. Films containing 5% leaf powder achieved antioxidant levels of 3.95 μmol Trolox g^−1^, 10.60 μmol TEAC g^−1^, and 0.97 mg GAE g^−1^ in 2,2-diphenyl-1-picrylhydrazyl (DPPH), 2,2′-azino-bis(3-ethylbenzothiazoline-6-sulfonic acid) (ABTS), and total phenolic assays, respectively, demonstrating in vitro antioxidant capacity and indicating potential for subsequent validation in lipid-containing foods. In addition, light acts as a catalyst for oxidation in photosensitive foods such as dairy products and oils. Many natural active substances inherently absorb ultraviolet light. Incorporating them into transparent hydrogel substrates can filter harmful ultraviolet radiation while maintaining packaging visibility, thereby physically blocking the energy source that initiates oxidation [82,83]. Wang et al. [84] introduced halloysite nanotubes (HNT) into a chitosan (CS) matrix to prepare CS/HNT composite films with both ethylene-scavenging and oxygen-barrier properties, blocking ultraviolet energy that initiates lipid oxidation and pigment degradation. As shown in Figure 7c–f, when the HNT content was 5 wt.%, the tensile strength of the composite film increased by 169.9%, oxygen permeability decreased by 52.94%, and ethylene adsorption capacity increased to five times that of the pure CS film, significantly extending the room-temperature shelf life of bananas and cherry tomatoes.

Swelling and release results should be interpreted together because water uptake increases polymer-chain mobility and may accelerate diffusion of low-molecular-weight active compounds. In the gelatin/chitosan/3-phenyllactic-acid system, the swelling ratio of GCP-1 was approximately twice that of the formulation without 3-phenyllactic acid, demonstrating that network chemistry altered both water absorption and antimicrobial-agent transport [28]. In the zein pseudolatex system, approximately 50% of the encapsulated carvacrol was released within 24 h [39], whereas halloysite nanotube encapsulation limited carvacrol release to 33.8% after 25 days at 40 °C [74]. In electrospun SPI/PVA coatings, the β-carotene loading efficiency was 65%, of which 51% was effectively encapsulated, and annealing changed release in a fatty-food simulant from an initial burst toward a more sustained profile [85]. These studies illustrate that swelling-mediated diffusion, hydrophobic nanocapsule partitioning, mineral-nanotube confinement, and post-spinning annealing produce markedly different release timescales. Accordingly, claims of “controlled” or “sustained” release should report the release medium, temperature, duration, cumulative release, and fitted kinetic model wherever available.

In addition to antimicrobial and antioxidant functions, active-packaging materials should be capable of actively regulating the atmosphere within the package. Ethylene is a ripening hormone that promotes fruit and vegetable maturation and senescence. Ethylene scavengers reduce ethylene concentrations in packages through three mechanisms: chemical oxidation (e.g., potassium permanganate converts ethylene into CO_2_ and water), physical adsorption (e.g., zeolites and activated carbon capture ethylene), and catalytic photodegradation (e.g., TiO_2_ decomposes ethylene under ultraviolet light). Together, these methods interrupt the ethylene-induced ripening signaling chain, thereby delaying the respiratory climacteric and quality deterioration of fruits and vegetables [86]. Zhang et al. [87] blended anthocyanin-rich black plum peel extract (BPPE) and TiO_2_ nanoparticles into chitosan (CS) films to prepare composite films combining antioxidant activity, ethylene scavenging, antimicrobial activity, and pH-responsive color development. Owing to the synergistic effects of the three components, the CS-TiO_2_-BPPE film showed optimal water and oxygen barrier properties, UV shielding, mechanical strength, and antimicrobial effects against *E. coli* and *S. aureus*.

The available real-food studies also differ substantially in food type, storage temperature, control treatment, packaging configuration, and endpoint used to define acceptability. At 4 °C, the gelatin/chitosan/3-phenyllactic-acid hydrogel delayed deterioration of chicken breast relative to the corresponding control [28], whereas gelatin–sodium alginate films containing 1% beetroot-peel extract were evaluated with vacuum-packaged minced beef over 14 days at 4 °C and produced lower microbial and oxidation indices together with improved color and sensory retention [41]. For plant products, CA-HNT/zein films maintained cherry-tomato quality for up to 15 days at 23 °C [74], and coaxially printed nanocellulose labels containing anthocyanins and 1-MCP extended lychee storage by 6 days at 25 °C under the conditions of the original study [88]. Ti-doped CuO/gelatin films maintained tomatoes for up to 18 days at 40 ± 3 °C, whereas the unwrapped and conventional-film controls showed pronounced deterioration after approximately 4 days [36]. These outcomes demonstrate application-specific preservation effects but should not be interpreted as a universal ranking of packaging performance because the foods, controls, storage environments, and spoilage criteria were different.

### 4.3. Intelligent Response Functions

Traditional “shelf-life” labels provide only time-based estimates and cannot reflect the actual condition of food during transportation and storage. Intelligent-packaging technology senses physical, chemical, or biological signals from food or the environment and converts them into color changes visible to consumers, thereby enabling real-time, nondestructive monitoring of food freshness, ripening status, and storage history. Freshness monitoring generally evaluates quality deterioration through indirect indicators such as pH, volatile metabolites, gas composition, and cumulative temperature exposure. By contrast, food safety monitoring requires the specific detection of biological or chemical hazards, such as pathogenic microorganisms, microbial toxins, allergens, or contaminants, together with validated detection limits and decision thresholds. In natural-polymer hydrogel systems, intelligent responses mainly rely on pH-responsive color changes, gas sensing, and temperature-memory mechanisms.

**Figure 8:**
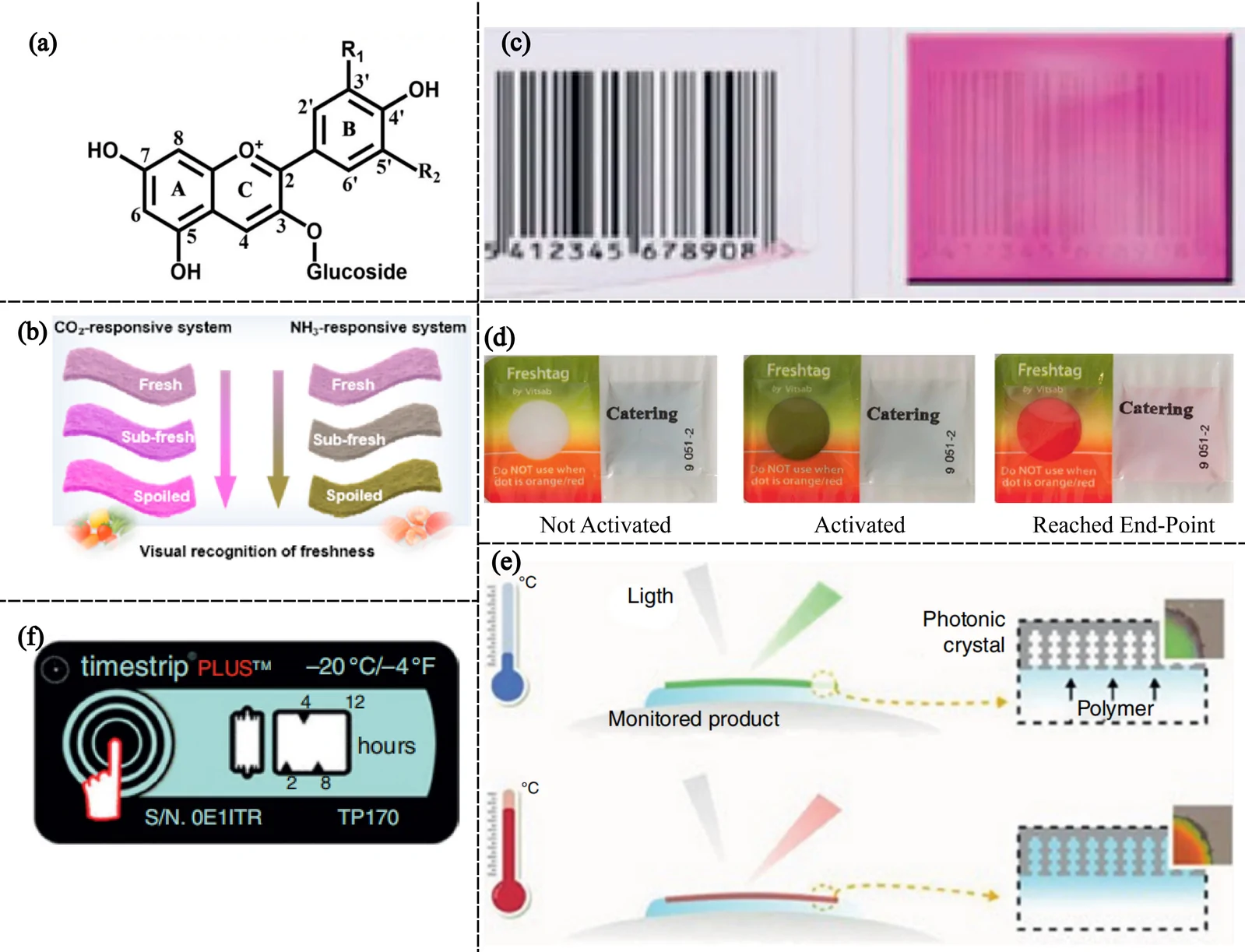
Colorimetric freshness indicators and time–temperature indicators for food-quality monitoring. (**a**) General chemical structure of anthocyanins and (**b**) pH-dependent color response to alkaline volatile compounds associated with protein deterioration. Reproduced with permission [91]. Copyright 2026 Elsevier. (**c**) Commercial TRACEO time–temperature indicator. (**d**) Colour change in active and expired Check Point TTI. (**e**) An indicator of time and temperature based on the optical response of photonic crystals impregnated with polymers. (**f**) Commercial time-temperature indicator (Timestrip PLUS) for monitoring cumulative temperature exposure. Reproduced with permission [110]. Copyright 2024 Wiley.

Anthocyanins are natural, edible, degradable, and capable of color changes over a wide pH range (from red to green/yellow at pH 1–14), resulting in readily observable color changes. Their basic chemical skeleton is shown in Figure 8a. Such color changes reflect alterations in the chemical environment surrounding the food and therefore provide an indirect indication of freshness rather than direct identification of pathogenic microorganisms or confirmation of food safety. Anthocyanins respond primarily to changes in the local acid–base environment. In protein-rich foods, ammonia and volatile amines can increase local pH and produce a visible color response. Hydrogen sulfide is a spoilage-related volatile compound but should not be described as an alkaline gas or assumed to generate the same anthocyanin response without compound-specific validation, as shown in Figure 8b. At the same time, their antioxidant and antimicrobial activities can delay food oxidation and microbial growth and extend shelf life. Compared with synthetic indicators, anthocyanins are widely available, derived from natural sources, capable of providing visible pH-dependent responses, and combine monitoring and preservation functions, making them ideal multifunctional natural packaging materials [89,90,91]. Ahmed et al. [92] blended anthocyanins with chitosan-polyethylene oxide (CS-PEO) and used electrospinning for batch preparation of nanofibrous membranes, exploiting their pH-sensitive color change as an “indicator.” When food spoilage generates alkaline substances such as volatile amines, the local pH of the membrane rises and the anthocyanins immediately change from red/purple to blue/green, allowing visual assessment. The degree of membrane color change is positively correlated with the concentration of spoilage gases, enabling instrument-free, real-time, visual detection of deterioration. In addition to anthocyanins, natural pigments such as curcumin [93,94,95], alizarin [96,97], betalains [98], chlorophyll [99], and carotenoids [100,101,102] have also been widely used to construct pH-responsive intelligent packaging materials for quality and freshness monitoring.

In addition to indirect detection through pH changes, some intelligent hydrogels can directly capture specific gas molecules, such as oxygen, carbon dioxide, and ammonia [103]. Food spoilage or fermentation releases CO_2_. Optical or electrochemical CO_2_ indicators convert changes in gas concentration into visible color changes or electrical signals. After calibration for a specific food product, packaging configuration, and storage condition, deviations from an established CO_2_ range can indicate changes in respiration, fermentation, or spoilage activity. Compared with conventional testing, this CO_2_ indication method is inexpensive, printable, and requires no specialized equipment; consumers can determine food freshness simply by observing the color change [104,105]. Zampino et al. [106] incorporated a long-chain imidazolium ionic liquid (IL) with a melting point of approximately 50 °C into a biobased polyether-polyamide copolymer film, using the IL’s solid-liquid phase transition to impart a temperature-responsive abrupt change in CO_2_ permeability. When the temperature rose above the IL melting point, film permeability increased sharply, and when cooled, it decreased sharply, forming a reversible CO_2_ valve. By monitoring the magnitude of the abrupt change in CO_2_ permeability across the film, temperature fluctuations within the package could be sensed in real time, and changes in CO_2_ concentration caused by spoilage gas production could be indicated indirectly. This mechanism therefore represents indirect monitoring of storage conditions and quality evolution rather than direct detection of food safety hazards.

Cold-chain transportation is essential for ensuring the quality of fresh food. Once an abnormal temperature increase occurs, food quality has already been damaged even if the food is subsequently refrozen [107]. The time-temperature indicator (TTI) shown in Figure 8c–f is a small intelligent label attached to food packaging that irreversibly records the cumulative “time-temperature” history experienced by a product throughout distribution through changes in color or form. It uses chemical, enzymatic, microbial, or other reactions to visualize the effect of temperature on food deterioration, allowing consumers to assess cold-chain integrity and the remaining acceptable shelf life [108,109,110]. A TTI records temperature history rather than directly measuring the current microbial or toxicological status of the food. Its response must therefore be calibrated against the deterioration kinetics of the target product before it can support quality-based acceptance decisions. In addition, TTIs can support timely adjustment of food-storage temperatures and support shelf-life management, and potentially reduce avoidable waste after product-specific calibration [111].

Intelligent response systems can convert multidimensional deterioration signals, including pH, volatile metabolites, package-gas composition, and cumulative time–temperature exposure, into visible color, pattern, or electrical changes. These responses provide information at different levels: pigment- and gas-responsive systems primarily indicate freshness or quality changes, whereas TTIs reflect storage history and cold-chain integrity. Direct food safety monitoring represents a more stringent category and requires selective recognition of a defined hazard, quantitative calibration, appropriate detection limits, and validation in the relevant food matrix. By establishing quantitative relationships among sensor response, storage conditions, microbial counts, chemical deterioration indices, and sensory acceptability, intelligent hydrogel-based packaging can support real-time quality evaluation, supply-chain management, and evidence-based shelf-life decisions.

## 5. Forming and Processing Technologies for Hydrogel-Based Packaging

To convert functionalized natural-polymer formulations into practical packaging forms (films, coatings, or containers), forming technologies matched to the rheological characteristics of the substrates must be used. Unlike conventional petroleum-based plastics, which rely on high-temperature melt processing, the processing of biobased materials must balance the protection of thermal stability (preventing deactivation of active components) with production efficiency. Accordingly, the principal methods are compared below in terms of their effects on material structure, functional retention, scalability, and environmental burden, as summarized in Table 2.

### 5.1. Solution Casting

Solution casting is the most fundamental and widely used wet film-forming process and is particularly suitable for laboratory research and the production of high-value-added active packaging. In this process, polymers (such as sodium alginate, PBAT, or PLA) and functional components (such as cinnamic acid and curcumin) are stirred and dissolved in a solvent (water, chloroform/dichloromethane, or dilute acid/alkali). After ultrasonic degassing, a measured amount is uniformly spread on a flat substrate and dried at room temperature or 40–60 °C to remove the solvent and form a film [112,113]. A technical advantage of this process is that film formation generally occurs at room temperature or at temperatures no higher than 90 °C, effectively preventing degradation of heat-sensitive components such as anthocyanins, enzymes, and essential oils. In addition, the process is simple and requires inexpensive equipment; film thickness and active-agent loading can be precisely controlled; and nanofillers or active components can readily be incorporated in situ to achieve multifunctionality [114,115]. Wu et al. [116] used solution casting to construct a functionally partitioned polylactic acid (PLA)-based composite film (PLA/CA) containing chitosan (CS) and alizarin (AL). The outer PLA/CS ring provided antimicrobial preservation, while the central PLA/AL region used the pH-responsive color change of alizarin to visualize chicken freshness. The film combined antioxidant activity, UV shielding, and reversible colorimetric response, enabling real-time monitoring of total volatile basic nitrogen (TVB-N) and pH changes and extending shelf life by 1 day. However, this method requires a long drying period (often dozens of hours), places a heavy burden on solvent recovery (chloroform and dichloromethane), and is difficult to implement continuously over large film areas. Film uniformity is affected by environmental humidity, and shrinkage and cracking readily occur at high solid contents. Mechanical strength is generally lower than that of extruded films, requiring subsequent lamination or crosslinking to meet mechanical requirements during transportation, which constrains large-scale industrial application [117,118].

Although casting is valuable for formulation screening, heat-sensitive components, and small-batch functional films, conventional batch casting has limited industrial productivity because a large amount of solvent must be removed from a relatively thin wet layer. Continuous tape casting, roll-to-roll coating, controlled convective drying, and solvent-recovery systems can improve scale-up, but they require control of wet-film thickness, drying gradients, edge effects, and residual solvent. Consequently, the environmental and economic performance of casting should be assessed using solvent consumption and recovery, drying energy per unit film area, solids loading, production yield, and line speed.

### 5.2. Thermoplastic Extrusion

In conventional film extrusion, a plasticized polymer formulation is continuously transported, mixed, heated, and pressurized by one or more screws before being shaped through a flat or annular die. This method is primarily applicable to thermoplastic or thermoplasticized hydrogel-forming polymers and to hydrogel-derived films rather than to fully hydrated bulk hydrogels. Starch, PVA, modified polysaccharides, and some protein-based formulations can be processed after water, glycerol, or other plasticizers reduce intermolecular interactions and increase chain mobility. The combination of heat and shear disrupts hydrogen bonding and ordered domains, promotes starch gelatinization or polymer melting, and disperses fillers within the matrix. Subsequent cooling, moisture equilibration, and polymer recrystallization determine the final network density, phase morphology, mechanical properties, and gas-barrier performance.

Ge et al. [119] prepared multilayer blown films containing a thermoplastic-starch/PVA barrier layer between LDPE outer layers. Certain TPS/PVA compositions provided oxygen-barrier and mechanical performance comparable to conventional barrier-layer materials. The study demonstrates the compatibility of hydrophilic polymer blends with continuous co-extrusion, but the LDPE-containing multilayer should not be presented as a fully bio-based or readily biodegradable hydrogel package. Estevez-Areco et al. [120] prepared a ZnO-containing thermoplastic cassava-starch layer by flat-die extrusion and combined it with an electrospun PVA layer containing rosemary polyphenols. Extrusion produced the load-bearing and antimicrobial layer, whereas electrospinning protected and delivered the antioxidant component. The bilayer increased mechanical properties and reduced water-vapor permeability relative to the individual structures. This study provides a useful processing–structure–function example: extrusion generated a comparatively dense structural substrate, while the porous electrospun layer supplied high active-agent loading. Wongphan and Harnkarnsujarit [121] used cast extrusion to prepare hydroxypropyl thermoplastic cassava-starch films containing agar and maltodextrin. The degree of starch substitution and the dispersion of agar-rich domains affected processability, surface morphology, mechanical properties, and water-vapor and oxygen permeability. This finding demonstrates that extrusion performance is governed not only by processing temperature but also by molecular substitution, plasticization, phase compatibility, and the microstructure formed during cooling. Gao et al. [122] further demonstrated the functional potential of blown extrusion by using starch as a matrix to regulate the release of propyl gallate. The extrusion-derived films provided sustained antioxidant release and delayed oxidative rancidity in peanut butter, showing that extrusion processing can influence not only film formation but also the subsequent transport and release behavior of active compounds.

Compared with laboratory solution casting, extrusion offers continuous production, higher throughput, more reproducible thickness, and compatibility with established film-blowing, flat-die, and multilayer-processing equipment. The scalability of extrusion is further illustrated by Iacovone et al. [123], who fabricated multifunctional cassava-starch bilayers containing basil extract and eco-friendly synthesized silver nanoparticles using an extrusion-based process. The multilayer architecture spatially separated antioxidant/UV-protective and antimicrobial functions, while maintaining the processing advantages of a continuous manufacturing route. This example highlights the potential of extrusion for integrating multiple functions without relying on a single homogeneous formulation. Its limitations include the thermal and shear sensitivity of natural polymers and active agents, restricted processing windows, moisture-dependent rheology, die fouling, start-up waste, and the need for controlled conditioning after extrusion. Heat-sensitive pigments, essential oils, enzymes, and volatile antimicrobials may require encapsulation, downstream coating, post-extrusion loading, or multilayer designs rather than direct incorporation into the melt. Extrusion should therefore be regarded as a promising scale-up route for thermoplastic hydrogel-derived formulations, not as a universal processing method for all hydrogel systems.

### 5.3. Electrospinning

Electrospinning is a simple and efficient technique that stretches polymer solutions or melts into nanoscale fibers under a high-voltage electric field. By controlling equipment and process parameters, this method can encapsulate natural or synthetic polymers and functional active substances with antimicrobial, antioxidant, or other properties into nanofibrous membranes for food packaging, thereby extending shelf life and improving safety [85,124]. Fiber formation is controlled by solution viscosity, conductivity, surface tension, polymer entanglement, flow rate, applied voltage, tip-to-collector distance, temperature, and humidity. These variables determine fiber diameter, bead formation, pore size, and fiber orientation, which in turn regulate mechanical integrity, gas and moisture transport, active-agent loading, and release kinetics.

**Figure 9:**
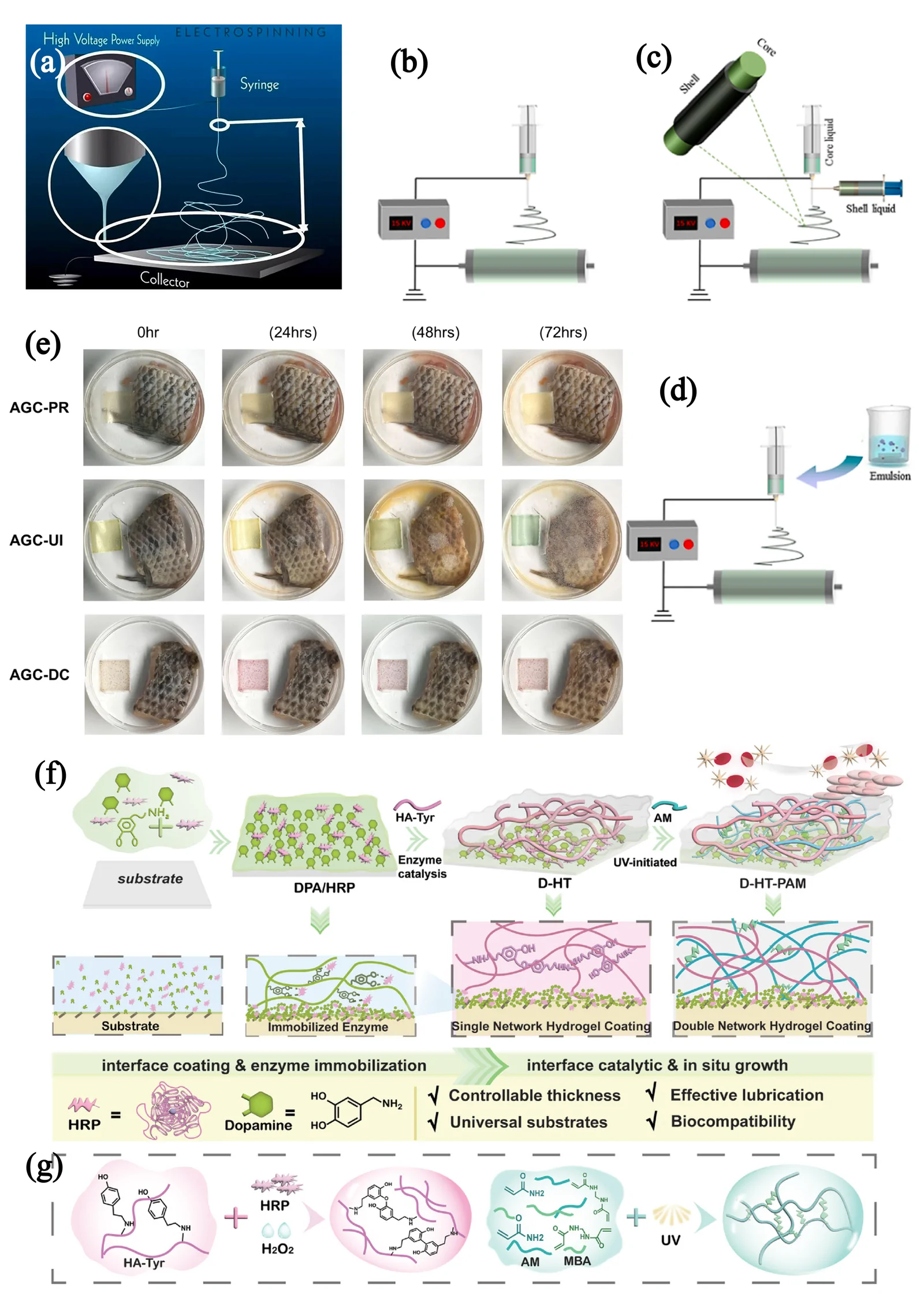
(**a**) Schematic diagram of an electrospinning setup. (**b**) Uniaxial electrospinning. (**c**) Coaxial electrospinning. (**d**) Emulsion electrospinning. Reproduced with permission [126]. Copyright 2024 Elsevier. (**e**) Colorimetric response of a 3D-printed pH sensor to TVB-N generated during packaged tilapia spoilage at room temperature (25 °C). Reproduced with permission [131]. Copyright 2024 American Chemical Society. (**f**) Schematic illustration of the enzyme-immobilized surface-catalyzed cross-linking (EISCC) strategy for constructing double-network D-HT-PAM hydrogel coatings on diverse substrates. (**g**) Schematic illustration of the formation of HA-Tyr-derived hydrogel networks through HRP/H_2_O_2—_mediated crosslinking and subsequent network construction. Reproduced with permission [133]. Copyright 2024 Wiley.

Pinheiro et al. [125] used conventional emulsion electrospinning to encapsulate hydrophobic β-carotene in SPI:PVA composite nanofibers and directly coated the fibers onto the surface of a PHA packaging film, regulating release behavior through annealing. The results showed a fiber loading efficiency of 65%, of which 51% was effectively encapsulated within the fibers. Annealing significantly improved coating adhesion and changed β-carotene release in simulated fatty foods from rapid burst release to sustained release, providing a feasible approach for developing antioxidant active packaging. As shown in Figure 9a–d, electrospinning can prepare nanoscale fibers under relatively mild thermal conditions and can efficiently encapsulate heat-sensitive active components for controlled release [126]. However, organic solvents are required; the fibers have low mechanical strength and are fragile, and single-needle throughput is low; consequently, large-scale production still faces process and equipment bottlenecks [127].

Electrospinning should not be described simply as an inexpensive and efficient process. Although many systems operate at ambient temperature, solution electrospinning may require volatile organic solvents, solvent extraction and recovery, controlled ventilation, and high-voltage equipment. Aqueous formulations reduce solvent-related concerns but often require PVA, PEO, or another carrier polymer to provide sufficient chain entanglement. Single-needle systems have low productivity, while multi-needle and needleless configurations can improve throughput at the expense of greater process complexity and possible variation in fiber diameter and deposition uniformity. Electrospinning is therefore currently most appropriate for thin functional coatings, active interlayers, and high-value indicator components rather than as a stand-alone high-volume structural-film process.

### 5.4. 3D Printing Technology

3D printing is an advanced manufacturing technology characterized by customization, low cost, and material diversity, and it can precisely construct complex structures and multifunctional components. It can use food-grade, degradable biobased materials (such as chitosan and PLA) and integrate sensors and indicators to intelligently monitor food freshness-related or storage-condition signals [88,128,129]. Tang et al. [130] used a curcumin/oregano-oil Pickering emulsion as functional ink and prepared a pH-sensitive composite film by 3D printing for real-time monitoring of fish freshness. The resulting film had both antioxidant and antimicrobial properties and underwent a visible yellow-to-purple color change in response to the release of volatile amines during storage, enabling visual freshness indication. Popoola et al. [131] developed 3D-printed nanocellulose-based pH sensors for food-freshness monitoring. As shown in Figure 9e, the sensor exhibited progressive color changes in response to TVB-N generated during the spoilage of packaged tilapia at 25 °C, demonstrating the feasibility of integrating 3D-printed hydrogel-based structures with visual freshness indication. Barekat et al. [132] used soy protein isolate as the base material and incorporated 0–5 wt.% Chlorella powder by 3D printing to prepare edible films, systematically optimizing nozzle diameter and printing speed. Under conditions of 3% algal powder, a 0.72-mm nozzle, and a printing speed of 20 mm/s, the films exhibited an increase in tensile strength of more than 40%, a reduction in water vapor permeability of more than 40%, and a doubling of DPPH antioxidant activity, yielding a bio-based printed film with improved mechanical, barrier, and in vitro antioxidant properties with a dense structure, enhanced antioxidant activity, and precise shape.

3D printing provides a versatile platform for the fabrication of geometrically customized and spatially organized food-packaging components. Its layer-by-layer deposition enables the controlled distribution or physical separation of active agents, freshness indicators, and structural materials, thereby facilitating the integration of preservation and monitoring functions within a single packaging system. The compatibility of extrusion-based printing with hydrogel-forming and bio-based formulations further supports its application in customized indicator labels, localized sensors, and multifunctional packaging components. Future development should focus on optimizing ink rheology, interlayer adhesion, mechanical and barrier properties, printing efficiency, and post-processing requirements, while evaluating environmental performance in relation to material utilization, energy consumption, production scale, and end-of-life conditions. These advances will determine the transition of 3D printing from laboratory-scale functional prototypes toward reproducible and scalable food-packaging applications.

### 5.5. In Situ Formation

The in situ method triggers a sol-gel transition of a polymer solution at the food-packaging interface. Because the coating forms on the target surface, this approach can provide conformal coverage of irregular geometries and stronger interfacial adhesion than separately prepared films. Reaction rate, solution wettability, polymer diffusion, and crosslinker transport determine coating thickness, network gradients, defect formation, and adhesion.

As shown in Figure 9f,g, it generates a tough, functional three-dimensional-network hydrogel coating on the surface of a substrate of any shape, thereby isolating food from external oxygen, water vapor, and microorganisms [133,134]. Ma et al. [135] used an in situ method to grow bacterial cellulose (BC) on cotton fibers, forming a sandwich-structured BCF film, and then vacuum-adsorbed curcumin to prepare an active intelligent composite film. The effects of the BCF structure on film mechanics, thermal stability, antioxidant activity, and pH-responsive color change were systematically investigated. The results showed that the cotton-fiber interlayer significantly increased curcumin loading in the BCF film, which changed from bright yellow to brown over pH 7–10 and exhibited higher antioxidant activity than the pure BC film, thereby combining antioxidant function with visual freshness indication. In situ synthesis of hydrogels can rapidly form films at packaging interfaces, simultaneously converting fruit-peel waste into antimicrobial and antioxidant components, extending shelf life at room temperature, and allowing biodegradation [136]. However, the reaction requires precise control; nonuniform film formation results in weak oxygen and moisture barriers and low mechanical strength, while batch-to-batch variations in raw materials readily reduce storage stability [137]. In addition, the in situ method cannot release active substances on demand in response to changes in the packaging microenvironment; that is, it lacks a stimulus-responsive “switch,” resulting in fixed and uncontrollable sustained-release behavior [75].

### 5.6. Food-Contact Compliance, Industrial Translation, Economic Feasibility, and Life-Cycle Sustainability

Food-contact compliance should be treated as an integral design requirement for hydrogel-based packaging rather than as a property inferred solely from the biological origin or reported biocompatibility of the constituent polymers [138]. Although cytotoxicity assays provide useful preliminary information, they do not independently establish the safety of a finished food-contact material. A safety assessment should consider the complete formulation and manufacturing process, including the chemical identities and use levels of polymers, crosslinkers, plasticizers, catalysts, solvents, active compounds, and sensing agents, as well as residual monomers, unreacted crosslinkers, reaction by-products, impurities, and degradation products [139]. Chemical modification or network formation may alter the migration behavior and toxicological profile of otherwise familiar food-grade components; consequently, compliance should be evaluated for the finished material under its intended conditions of use rather than inferred from the regulatory status of individual starting materials.

Regulatory assessment is jurisdiction-specific [140]. In the European Union, hydrogel-based packaging intended for food contact must comply with the general safety requirements of Regulation (EC) No 1935/2004 and the good manufacturing practice requirements of Regulation (EC) No 2023/2006, as amended. For packaging materials or layers that fall within the scope of plastics legislation, the specific requirements of Commission Regulation (EU) No 10/2011, including its current amendments, should also be considered. Systems intentionally designed to release or absorb substances may additionally fall within the scope of Regulation (EC) No 450/2009 on active and intelligent materials and articles. In addition, Regulation (EU) 2025/40 on packaging and packaging waste applies from 12 August 2026 and introduces broader packaging sustainability requirements, including restrictions on PFAS in food-contact packaging. In the United States, the regulatory status of each food-contact substance and its intended conditions of use should be established through the applicable authorization, notification, or exemption pathway. Consequently, compliance should be determined for the complete packaging system and its intended application rather than inferred solely from the natural origin or prior food use of individual constituents.

The swelling and water-mediated transport characteristics of hydrogel networks may facilitate the diffusion of low-molecular-weight constituents. Overall and specific migration should therefore be examined using food simulants or representative foods selected according to the expected food composition, contact duration, temperature, surface-area-to-food ratio, and single- or repeated-use conditions. Film thickness, hydration state, crosslinking density, and changes in network integrity during contact should also be reported because migration from a dry film may differ substantially from that of the same material after swelling. Analytical assessment should distinguish intentionally released active substances from unintended migrants and should, where relevant, quantify residual crosslinkers, oligomers, plasticizers, colorants, essential-oil constituents, metal ions, and degradation products. Migration results should subsequently be combined with estimates of dietary exposure and toxicological information to determine whether the anticipated exposure is acceptable under the proposed use conditions [141,142].

Nano-enabled hydrogel packaging requires additional consideration because the incorporation of Ag, ZnO, TiO_2_, CuO, carbon dots, or other nanoscale components may alter both material performance and exposure pathways. Low total migration or low cytotoxicity alone does not establish nanospecific safety [143]. Appropriate evaluation should characterize particle size and size distribution, morphology, surface chemistry, aggregation or agglomeration state, dissolution behavior, and transformation within the polymer network and food matrix. Migration studies should distinguish particulate migration from the release of dissolved ions and should consider the effects of swelling, mechanical abrasion, repeated handling, ultraviolet exposure, temperature, and material ageing. Where nanoscale exposure cannot be excluded, hazard characterization should address nanospecific endpoints and the behavior of the material following ingestion, in accordance with applicable risk-assessment guidance [144,145].

The industrial translation of hydrogel-based packaging requires the simultaneous consideration of material performance, manufacturing compatibility, food-contact compliance, economic feasibility, and environmental consequences [146]. Laboratory-scale improvements in tensile strength, barrier performance, antimicrobial activity, or indicator response do not independently establish manufacturing readiness. Translation should instead be evaluated using application-specific performance thresholds, batch-to-batch reproducibility, film-thickness uniformity, active-agent retention, storage stability, processing yield, production rate, and compatibility with converting operations such as coating, printing, lamination, sealing, and winding [27]. Pilot-scale production under controlled temperature and humidity is therefore an essential intermediate stage between laboratory formulation and commercial implementation.

Economic feasibility should be assessed on the basis of the complete packaging system rather than polymer price alone. Relevant contributors include feedstock collection and purification, polymer modification, crosslinkers, plasticizers, active or sensing compounds, solvent and water use, drying or curing energy, equipment investment, labor, material losses, quality control, and regulatory testing. Techno-economic analysis of an agro-industrial-residue-derived PLA/PHB packaging film demonstrated that feedstock availability, production capacity, conversion efficiency, and plant scale can substantially affect the minimum selling price and investment performance [147]. Although the material system differs from hydrogel packaging, the analysis provides a transferable methodological basis for evaluating the economic feasibility of biomass-derived packaging materials.

Full-life-cycle sustainability requires defined system boundaries and quantitative evaluation criteria. Raw-material assessment should include feedstock origin, land and water requirements, extraction and purification yields, chemical consumption, and the utilization of agricultural or food-processing by-products. Manufacturing assessment should quantify solvent recovery, water consumption, energy demand, production efficiency, scrap generation, emissions, and the environmental effects of crosslinkers, nanofillers, and functional additives. Comparative environmental assessments of chitosan- and cassava-starch-based packaging have shown that renewable polymer origin does not eliminate environmental burdens associated with acid-mediated extraction, additives, agricultural production, and energy-intensive film manufacture [148,149]. During use, the environmental burden of the packaging should be evaluated together with its capacity to reduce food deterioration and waste. End-of-life assessment should distinguish recycling, industrial or home composting, biodegradation under defined conditions, dissolution, disintegration, and landfill disposal, while also considering degradation products, ecotoxicity, and the persistence or migration of nanoscale components.

Life-cycle comparisons should use equivalent packaging functions rather than equal material masses. Appropriate functional units may include the quantity of packaging required to protect a specified amount of food for a defined storage period or to achieve a defined reduction in food loss. Relevant indicators include greenhouse-gas emissions, cumulative energy demand, water consumption, land use, eutrophication, toxicity-related impacts, material circularity, and end-of-life recovery. Such quantitative evaluation can identify trade-offs among renewable feedstocks, processing intensity, preservation performance, and disposal behavior and can support the development of hydrogel packaging systems with demonstrable environmental advantages.

**Table 2:** Qualitative comparison of solution casting, thermoplastic extrusion, electrospinning, three-dimensional printing, and in situ formation in terms of resulting structure, principal processing advantage, main limitation, and relative scale-up potential for hydrogel-based and hydrogel-derived food-packaging materials.

Process Type	Resulting Structure	Principal Advantage	Main Limitation	Relative Scale-Up Potential
Solution Casting	Dense or phase-separated dried network	Broad formula-tion compati-bility; suitable for heat-sensitive compounds	Long drying time; thickness and drying gradients	Low–moderate
Thermo-plastic extrusion	Dense, ori-ented or multilayer thermoplastic structure	Continuous pro-cessing and high throughput	Thermal/shear degradation; restricted material window	High for thermoplasticized formulations
Electrospinning	Porous nanofibrous layer	High loading area and controllable release	Low intrinsic strength and limited single-needle throughput	Low–moderate
3D Printing	Patterned, layered or core–shell structure	Spatial control and customization	Slow printing; rheological constraints; anisotropy	Low for full films; moderate for labels
In Situ Synthesis	Conformal interfacial network	Strong substrate adhesion and coverage of complex surfaces	Reaction control, residual compounds and reproducibility	Moderate for coatings

## 6. Conclusions and Outlook

Hydrogel-based and hydrogel-derived materials provide tunable platforms for integrating barrier, active-preservation, and freshness-indication functions. Current evidence demonstrates application-specific potential, although performance, safety, manufacturability, and environmental benefits remain dependent on formulation and use conditions. This article critically reviewed recent advances in multifunctional hydrogels based on natural and synthetic polymers for food packaging, covering key aspects such as substrate selection, functional design strategies, network construction, forming and processing technologies, application validation, and industrial translation. Existing studies show that molecular-level chemical modification, nanofiller compounding, and multi-network construction have significantly improved the mechanical strength, water-vapor barrier performance, and environmental stability of hydrogels. The incorporation of antimicrobial agents, antioxidants, and responsive indicators has further enabled the regulation of microbial growth, oxidation, moisture transfer, and spoilage-associated signals. Real-food studies involving fruits, vegetables, aquatic products, and meat demonstrate that selected hydrogel films, coatings, absorbent structures, and indicator labels can delay quality deterioration or provide information on freshness under defined storage conditions. These findings establish the application potential of hydrogel-based packaging, particularly when its hydrated network or multifunctionality addresses requirements that are difficult to achieve using a passive packaging film alone.

The current body of evidence nevertheless consists predominantly of laboratory-scale formulations and application-specific proof-of-concept studies. Differences in film thickness, relative humidity, testing protocols, food composition, storage temperature, and packaging configuration also limit direct comparison among reported systems. Their suitability must be determined according to the required mechanical durability, moisture and gas barriers, active-agent stability, food-contact conditions, and storage environment of the target product. Translation toward practical application requires coordinated optimization of material performance, manufacturing reproducibility, safety, cost, and environmental behavior. The inherent hydrophilicity of many hydrogel networks facilitates swelling and active-compound transport but can reduce dimensional stability and moisture-barrier performance under high-humidity conditions. Hydrophobic modification, increased crosslinking density, and nanofiller reinforcement can improve water resistance and strength, although these changes may also affect flexibility, transparency, swelling-mediated response, and active-agent release. Future material design should therefore focus on balancing coupled properties rather than maximizing a single mechanical or barrier parameter.

Looking ahead, to achieve the rational design and resource-efficient and environmentally evaluated manufacturing of multifunctional hydrogel packaging materials, we believe the following directions warrant particular attention. (1) Multidimensional upgrading of intelligent response systems. Current pH-responsive indicators are mostly limited to natural pigments such as anthocyanins, whose color stability and response sensitivity still require improvement. Future research could integrate multiple response mechanisms—such as temperature, enzyme activity, and specific spoilage metabolites—within a single packaging system and combine them with Internet of Things technology to achieve real-time wireless monitoring of food quality, thereby advancing packaging from “visualization” to “communication.” (2) Biomimetic and composite materials design. Inspired by layered structures in nature, such as nacre, and gradient structures, such as bamboo, constructing ordered microstructures may help resolve the conflict among hydrogel strength, barrier performance, and toughness. Blending natural polymers with PVA, PLA, or other processable polymers may improve rheology, water resistance, and mechanical performance; however, the resulting material’s degradation behavior must be determined for the complete formulation under defined end-of-life conditions. (3) Quantitative life-cycle sustainability assessment. Future sustainability claims should be supported by comparative life-cycle assessments using functionally equivalent packaging units and clearly defined system boundaries. Such assessments should quantify the environmental burdens associated with raw-material acquisition, polymer modification, solvent and water consumption, drying or curing, packaging performance during use, and end-of-life treatment. The potential environmental benefits of reducing food loss should be included only when shelf-life extension has been validated under representative storage and distribution conditions. Meanwhile, biodegradation, disintegration, dissolution, mass loss, and compostability should be evaluated as distinct end-of-life outcomes using standardized methods and clearly specified environmental conditions. Such evidence will clarify the conditions under which hydrogel-based packaging can provide measurable functional and environmental advantages within circular material systems.

## Data Availability

No new data were created or analyzed in this study. Data sharing is not applicable to this article.
